# Assessment of reference genes at six different developmental stages of *Schistosoma mansoni* for quantitative RT-PCR

**DOI:** 10.1038/s41598-021-96055-7

**Published:** 2021-08-19

**Authors:** Gilbert O. Silveira, Murilo S. Amaral, Helena S. Coelho, Lucas F. Maciel, Adriana S. A. Pereira, Giovanna G. O. Olberg, Patricia A. Miyasato, Eliana Nakano, Sergio Verjovski-Almeida

**Affiliations:** 1 Laboratório de Parasitologia, Instituto Butantan, São Paulo, SP 05503-900 Brazil; 2 Departamento de Bioquímica, Instituto de Química, Universidade de São Paulo, São Paulo, SP 05508-900 Brazil

**Keywords:** Parasitology, Gene expression analysis

## Abstract

Reverse-transcription quantitative real-time polymerase chain reaction (RT-qPCR) is the most used, fast, and reproducible method to confirm large-scale gene expression data. The use of stable reference genes for the normalization of RT-qPCR assays is recognized worldwide. No systematic study for selecting appropriate reference genes for usage in RT-qPCR experiments comparing gene expression levels at different *Schistosoma mansoni* life-cycle stages has been performed. Most studies rely on genes commonly used in other organisms, such as *actin*, *tubulin,* and *GAPDH*. Therefore, the present study focused on identifying reference genes suitable for RT-qPCR assays across six *S. mansoni* developmental stages. The expression levels of 25 novel candidates that we selected based on the analysis of public RNA-Seq datasets, along with eight commonly used reference genes, were systematically tested by RT-qPCR across six developmental stages of *S. mansoni* (eggs, miracidia, cercariae, schistosomula, adult males and adult females). The stability of genes was evaluated with geNorm, NormFinder and RefFinder algorithms. The least stable candidate reference genes tested were *actin*, *tubulin* and *GAPDH*. The two most stable reference genes suitable for RT-qPCR normalization were Smp_101310 (*Histone H4 transcription factor*) and Smp_196510 (*Ubiquitin recognition factor in ER-associated degradation protein 1*). Performance of these two genes as normalizers was successfully evaluated with females maintained unpaired or paired to males in culture for 8 days, or with worm pairs exposed for 16 days to double-stranded RNAs to silence a protein-coding gene. This study provides reliable reference genes for RT-qPCR analysis using samples from six different *S. mansoni* life-cycle stages.

## Introduction

Along with malaria, schistosomiasis is one of the most important parasitic diseases due to its high impact on morbidity and mortality rates, affecting more than 230 million people in 76 different tropical and subtropical countries^1^. The three main species that cause schistosomiasis are *Schistosoma japonicum*, *S. haematobium* and *S. mansoni*, the latter being prevalent in Africa and Latin America. In America, it is estimated that *S. mansoni* infects 1–3 million people, and over 25 million live in risk areas, being Brazil and Venezuela the most affected^2^. Sexual dimorphism is one of the main characteristics of these blood flukes. Furthermore, blood flukes have a complex developmental life cycle, comprising at least seven different stages (eggs, miracidia, sporocysts, cercariae, schistosomula, adult males and adult females). Life-cycle progression also requires two hosts, the definitive mammalian and the invertebrate Mollusca host from the genus *Biomphalaria*, resulting in complex development and making schistosomiasis very hard to control and prevent^3^.

With the advent of the *S. mansoni* transcriptome^4^ and genome^5,6^ sequences, the field was open for a large number of transcriptomic studies reported in the last two decades, enlightening gene expression patterns in different life-cycle stages^7–13^, as well as in tissues^14,15^, organs^16^ and single-cells^17,18^. A new *S. mansoni* transcriptome and genome version 7 have recently become available^19^. In addition, we built a new transcriptome assembly^20^ that merged all known protein-coding genes^19^ with long non-coding RNAs expressed by the parasite, which were identified using 633 publicly available *S. mansoni* RNA-Seq libraries^20^. The way is paved, therefore, for further functional characterization of protein-coding and long non-coding RNA genes across the parasite life cycle. For all transcriptomic and functional gene studies, the expression levels of each gene of interest found in the large-scale analyses need to be first confirmed by a more specific method such as RT-qPCR before further functional studies are applied.

Reverse transcription-quantitative real-time polymerase chain reaction (RT-qPCR) relies on two different ways to quantify gene expression levels: absolute or relative quantification. Relative quantification is widely used because it is less sensitive to sample preparation, RNA quality, and cDNA synthesis. However, a careful selection of appropriate reference genes (also known as housekeeping genes) for adequate^21^ normalizations is required before relative quantification RT-qPCR is applied.

Reference genes chosen for RT-qPCR normalization are frequently associated with housekeeping functions such as glycolysis, respiration, cell transport, and cytoskeleton, with *actin*, *tubulin* and *GAPDH* being some of the most frequently used genes. Nonetheless, pieces of evidence pointing out that those genes may exhibit varying expression levels in different stages/tissues or conditions tested are emerging^22^. Thus, it is now globally accepted that it is necessary to search for stable reference genes in the condition that is being specifically tested^22^, and that more than one reference gene should be used in the RT-qPCR normalization steps^23^. In *S. japonicum,* the identification of reference genes that are not differentially expressed in four life stages of the parasite has been accomplished, suggesting PSMD4 (*26S proteasome subunit*), TPC2L (*longin-like protein*), and NDUFV2 (*core subunit of respiratory chain Complex I*) as the most stable genes^24^.

As for *S. mansoni*, specific reference genes have been pointed so far only to study pairing-dependent gene expression in males^25^. No report has used high-throughput data to select specific reference genes for correct normalization of RT-qPCR assays comprising samples from different life-cycle stages in *S. mansoni*. Noteworthy, about 8% (1164 genes) out of the 14,548 protein-coding genes in the *S. mansoni* genome v.7 is annotated as “Hypothetical protein” (949 transcripts) or “Uncharacterized protein” (215 transcripts). In this context, as a first step towards the characterization of an uncharacterized gene of interest, it is important to have its correct quantification by RT-qPCR among the different *S. mansoni* life-cycle stages using a set of reliable reference genes.

In the present study, we selected and tested a group of twenty-five candidate reference genes for RT-qPCR assays across six *S. mansoni* life-cycle stages. Our selection was based on their expression stability, which was detected by using publicly available data of *S. mansoni* RNA-Seq libraries^18,26–29^ and by using four different methods (DESeq2^30^, TMM/CPM^31^, UQ^32^, and TPM^33^) to normalize and compare the large-scale expression data across the different samples. In addition, eight reference genes used by previous publications for the normalization of data from at least three different life-cycle stages of *S. mansoni* were also selected. Altogether, 33 candidate reference genes were selected and tested for their expression stability in a series of RT-qPCR experiments with RNA samples extracted from six different *S. mansoni* developmental stages (eggs, miracidia, cercariae, 48-h-schistosomula, adult males and adult females). The geNorm, NormFinder and RefFinder algorithms were used for evaluation of the candidate genes’ stability. The most stable genes were identified and pointed as reliable reference genes for use in RT-qPCR analyses among six different *S. mansoni* stages. These reference genes were also tested under two other assay conditions, the first involving adult females maintained in culture for 8 days as unpaired worms or as paired female/male couples, the second involving adult worm pairs treated with dsRNAs to silence the protein-coding gene *EED* (*Embryonic Ectoderm Development*), a component of the histone modifying complex Polycomb Repressive Complex 2 (PRC2), which tri-methylates H3K27.

## Results

### Identification of reference genes for the study by RT-qPCR of gene differential expression across *S. mansoni* life-cycle stages

To identify reference genes suitable for the study by RT-qPCR of gene differential expression across life-cycle stages, we selected RNA-Seq libraries from previous works^18,26–29^ available at the Sequence Read Archive (SRA) databank. These libraries had to accomplish a minimum of 50% aligned reads, having at least 4 million aligned reads in each library (Supplementary Table S1). Since most of the *S. mansoni* RNA-Seq data available is from schistosomula, adult male and female stages, we prioritized the six RNA-Seq libraries from each of these stages with the highest numbers of aligned reads and the highest percentage of aligned reads. For miracidium, there was only one available library, while two libraries were available from sporocysts. Thus, those three libraries were analyzed as a single group of Miracidium/Sporocyst (M/S). Exceptionally, library SRR11248243 (from sporocysts) had a 24.65% read alignment rate but was kept in the analysis because it had more than 28 million reads aligned. For cercariae, just three libraries passed the two cut-offs of the number of aligned reads and percentage of aligned reads. Overall, 24 RNA-Seq libraries that comprised miracidium/sporocysts (n = 3), cercariae (n = 3), schistosomula (n = 6), adult males (n = 6) and adult females (n = 6) (Supplementary Table S1) were used in our analyses.

We next established a bioinformatics pipeline to analyze the selected RNA-Seq libraries and search for the best candidate reference genes (see “Methods”). Among the RNA-seq data analysis steps, normalization is crucial to accurately interpret the results of transcriptomic experiments. Thus, we used the following four different normalization methods: DESeq2^30^, Trimmed mean of M values (TMM.CPM)^31^, Upper Quartile (UQ)^32^ and Transcripts per Million (TPM)^33^. The expression values for all *S. mansoni* transcripts in each tested normalization method are provided in Supplementary Tables S2–S5 for DESeq2, TMM.CPM, UQ, and TPM, respectively. A visualization platform for the expression values obtained with each normalization method was created and is available at https://verjolab.shinyapps.io/Reference-genes/.

**Figure 1:**
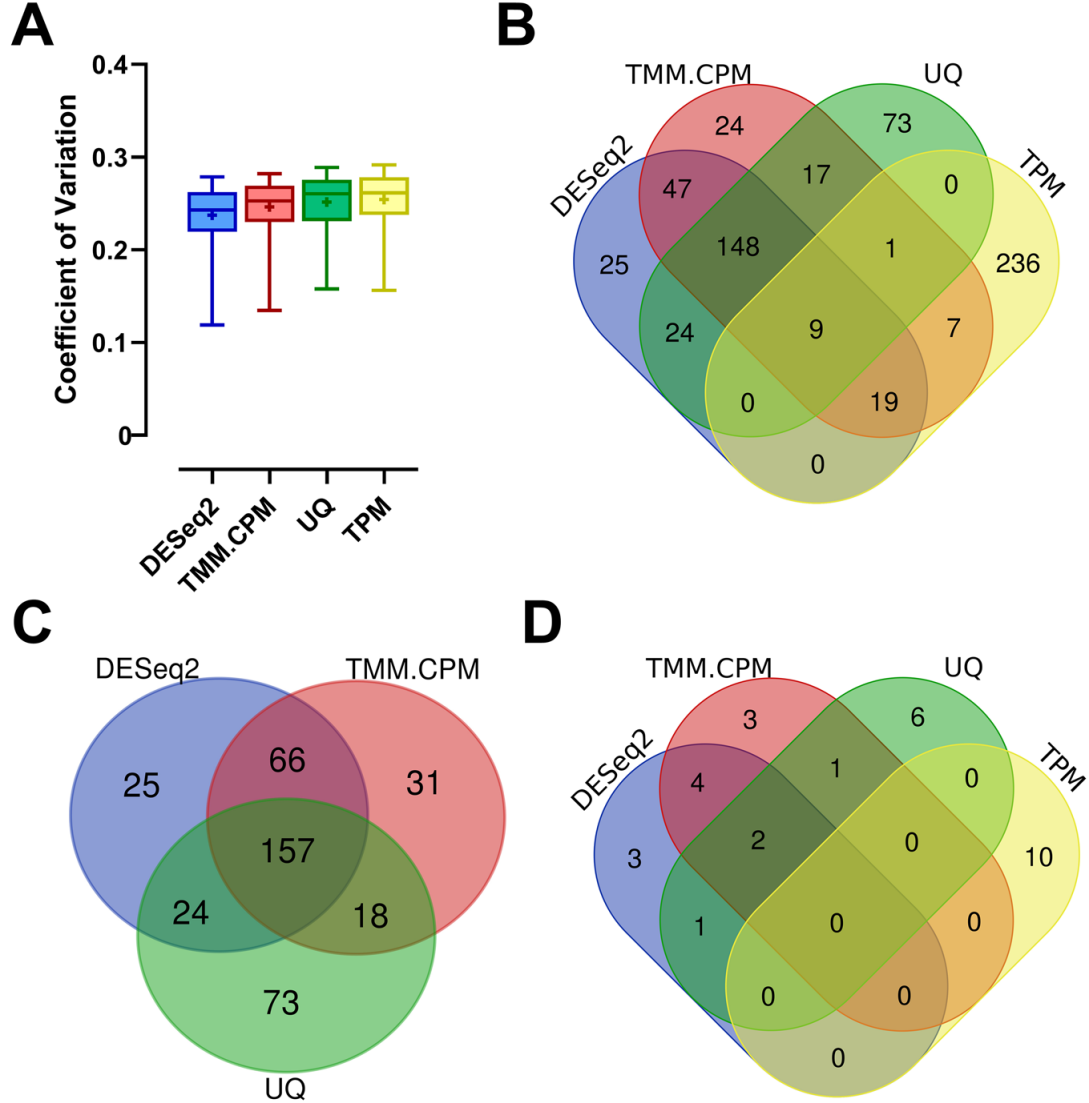
Transcriptome-wide analysis of the stability of candidate reference genes for quantitative RT-PCR assays across six life-cycle stages of *S. mansoni*. Twenty-four RNA-Seq libraries comprising six *S. mansoni* life-cycle stages were analyzed using four different normalization methods, namely DESeq2, TMM.CPM, UQ, and TPM. (**A**) Box plots of the coefficients of variation (CV), which were calculated based on the expression levels of the 272 genes with the lowest coefficients of variation, representing 2% of all genes detected in the RNA-Seq libraries, and analyzed with each of the four normalization methods indicated in the x-axis. The horizontal line represents the CVs’ median for each normalization method, while the + signal represents the mean. The boxes and whiskers represent the inter-quantile and min to max ranges, respectively. (**B**) Venn Diagram shows the number of genes and the overlap among the 272 most stable genes found in each of the four RNA-Seq normalization methods. (**C**) Venn Diagram showing the number of genes and the overlap among the 272 most stable genes in the DESeq2, TMM.CPM and UQ normalization methods. (**D**) Venn Diagram showing the number of genes and overlap among the ten most stable genes according to their CVs in each RNA-Seq normalization method tested. *UQ* upper quartile, *TMM* trimmed mean of M-values, *CPM* counts per million, *TPM* transcripts per million.

The first step to identify the most stable reference genes was to calculate for each of the 13,624 *S. mansoni* protein-coding genes (Smps) under analysis, the coefficient of variation across all libraries (CV = Standard Deviation of expression values across all libraries/Average of expression values across all libraries). The calculation was repeated for each of the four normalization methods. The analysis proceeded with the identification, for each normalization method, of the 272 Smps comprising the top 2% of all genes that showed the lowest CVs as the most stable ones. The means and medians of the coefficients of variation across these 272 Smps were similar for the four different normalization methods (Fig. 1A), with no significant differences. However, the group of 272 genes obtained in each normalization method was different, especially for the TPM normalization method. From all 272 Smps belonging to the 2% genes with the lowest CVs in the TPM normalization method, only 36 Smps (13% of them) were also present in at least one of the other three normalization methods (Fig. 1B).

A comparison among DESeq2, TMM.CPM and UQ normalization methods showed that 157 (or 58%) of the 272 Smps were common to all three normalization methods (Fig. 1C). Less than 27% of the 272 Smps in each method were not present in at least one of the other two normalization methods (Fig. 1C). With that in mind, we selected the top ten most stable Smps (those genes with the lowest CVs) from each normalization method to be compared against each other. We found out that at least 4 Smps identified in each of the three methods as the best candidate reference genes, out of top 10 genes, were common among DESeq2, TMM.CPM and UQ (Fig. 1D), resulting in a list of 20 unique Smps comprising all three normalization methods. Furthermore, all the ten most stable candidate reference genes identified with the TPM method did not overlap with any of the top 10 genes from the other three methods (Fig. 1D).

Thus, we selected for RT-qPCR assays the 20 candidate reference genes that comprise the set of unique genes in the overlap among the three lists of the top ten most stable genes in the analyses with DESeq2, TMM.CPM and UQ (Fig. 1D), plus 5 candidate reference genes comprising the top, most stable genes from the TPM normalization method. We also selected eight genes used in previous works as reference genes across different *S. mansoni* life-cycle stages, totalizing 33 candidate reference genes to be tested. Information regarding the eight previously reported reference genes, including the life-cycle stages in which they were measured, and if the gene expression change identified was validated by another method (such as western blotting or northern blotting), is reported in Supplementary Table S6.

**Figure 2:**
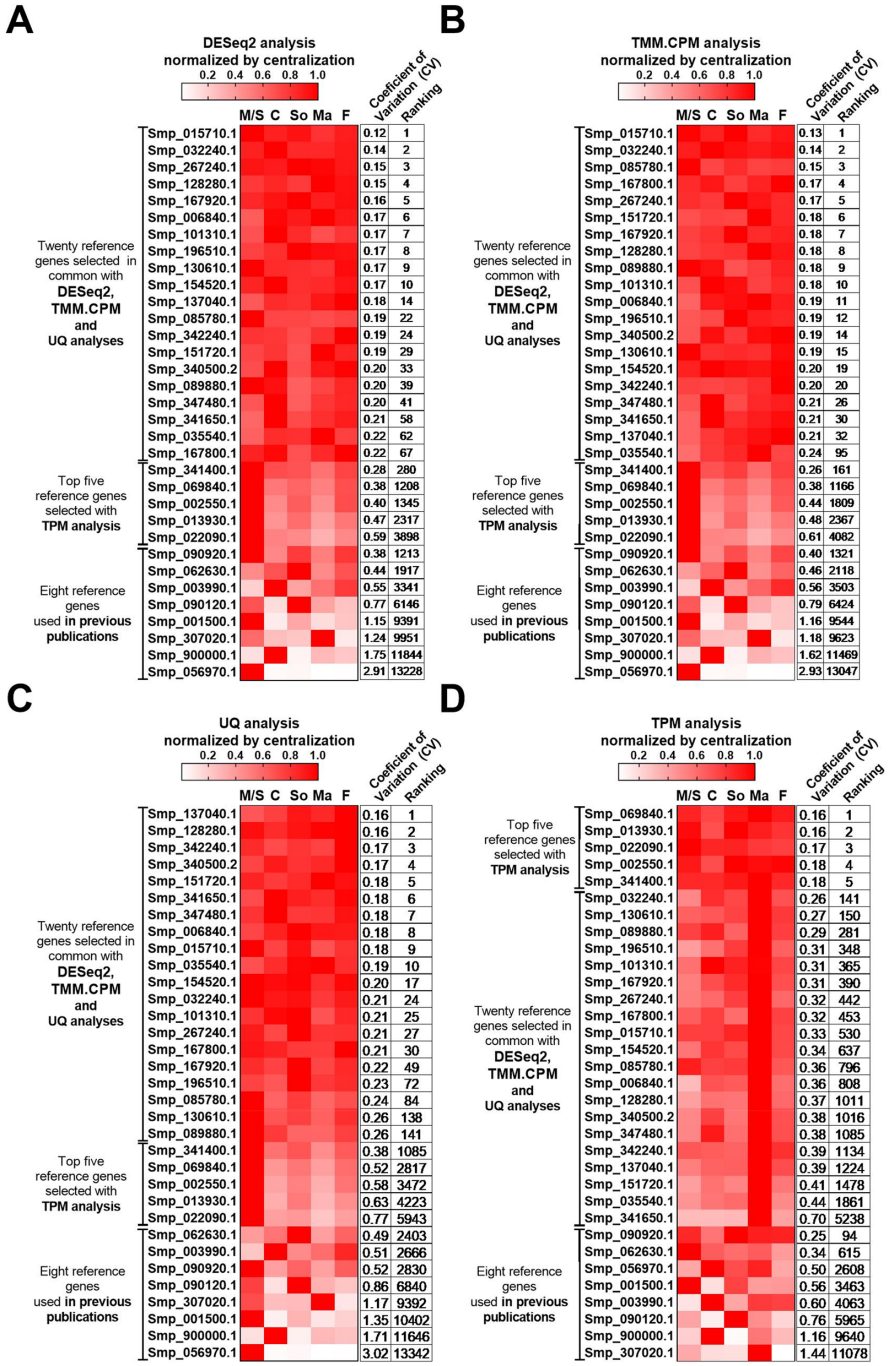
Expression patterns of candidate reference genes at different *S. mansoni* life-cycle stages. Twenty-five candidate reference genes with the lowest coefficients of variation of their expression values were selected to evaluate their stability by RT-qPCR assays. The selection was based on the analysis of 24 RNA-Seq libraries from six different *S. mansoni* life-cycle stages (see “Methods”). In addition, eight candidate reference genes commonly used in previous publications were also selected. The heatmaps were built based on the average expression values of each gene from all libraries used for each life cycle stage analyzed and were calculated for each RNA-Seq normalization method, namely (**A**), DESeq2; (**B**), TMM.CPM; (**C**), UQ; and (**D**) TPM. Smp stands for *S. mansoni* protein, and the codes are related to the gene annotation. *M/S* miracidia/sporocysts, *C* cercariae, *S* 48-h-schistosomula, *Ma* adult males, *F* adult females. The expression coefficient of variation and each gene's ranking across the different RNA-Seq normalization methods are reported on the two columns at right in each panel.

The ranking for all 33 selected candidate reference genes is presented in Fig. 2, along with a heatmap of their expression values determined by DESeq2 (Fig. 2A), TMM.CPM (Fig. 2B), UQ (Fig. 2C), and TPM (Fig. 2D) normalized by centralization (which represents the expression of that gene in each of the life-cycle stages). The Log_10_ transformed expression values for each of the 33 candidate reference genes across all analyzed libraries are shown in Supplementary Fig. S1 for each of the normalization methods used.

Pairs of primers were designed to amplify each of the 25 candidate reference genes selected based on our RNA-seq data analyses. Their amplification efficiencies were measured using a dilution series of cDNA from male and female *S. mansoni* adult worms. We also measured the primers' amplification efficiency for the other eight reference genes used in previous works. Amplification efficiencies for all the primer pairs were higher than 92.6%. Their RT-qPCR amplification characteristics are presented in Supplementary Table S7. Gene annotation of all 33 genes, primer sequences and their mean Cq values are given in Table 1.

**Table 1:** Information on the RT-qPCR amplification characteristics of the 33 candidate reference genes.

Gene product name	Smp Code^a^	Forward primer sequence (5′–3′)	Reverse primer sequence (5′–3′)	Mean Cq^a^	Refs.^a^
NCK-interacting protein with SH3 domain	Smp_128280.1	CTCTGCGATCTGTTCTCTTACTC	TGAGAAGTAGCAGTAAATGAGGC	21.7	This paper
6-phosphofructo-2-kinase/fructose-2,6-bisphosphatase 1	Smp_015710.1	TATGGAATTAGAACGACAAACATCAG	GCAGTCGGTGTTAATTTGAATACAG	27.2	
Histone H4 transcription factor	Smp_101310.1	AAGTCAACCGATCCAGTTCTAC	TCTGCTTGAACATGTGGTAAGG	22.5	
Mitotic-spindle organizing protein 2B	Smp_032240.1	GACCGTCCCAAATGATGTTG	TGACATCTGGACTGCAACC	20.2	
Ubinuclein-2	Smp_167920.1	TTTAGTGTCCAAGGCGGTAC	ACCTTTATTTGAGCAGTGGGAG	20.8	
Palmitoyltransferase ZDHHC3	Smp_267240.1	AAGCTTATCCAGATGGTAGCG	GGCTTGAAAAGATATGAACATGGTAG	19.8	
Protein UBASH3A homolog	Smp_151720.1	GGCGATGGGTCGTTCAGATT	GCAGTACGATCACAATAGCCT	20.9	
UPF0060 membrane protein ESA_01751	Smp_006840.1	GTCCTAAATGTCGTAATGTGGC	AAAATACACCTACCAAAAGCTGC	19.5	
39S ribosomal protein L10, mitochondrial	Smp_085780.1	CTGTTTTCTGAGCCTACTCCTG	CCTTGAGATGACAATGCAGC	26.6	
FAD-dependent oxidoreductase domain-containing protein 1	Smp_089880.1	ACTGGTTTTCATCCATTTCATACAAA	ACGAAAAACGTACTAGATCTATTGTTT	31.0	
Zinc finger CCHC domain-containing protein 7	Smp_167800.1	GATAACCGAACTCATTCTTTGTCTTAC	CGCTGTTTTATGGCCTTCTG	25.7	
Protein FAM60A	Smp_154520.1	ATGACCCATAATCCCGAAGTG	AGCAGCAGTATGAATGGTACG	20.5	
tRNA (guanine-N(7)-)-methyltransferase	Smp_130610.1	AAAGCATTGCGTGTGAAGC	CACGTTTAAAATGAGGGTCTGG	26.1	
Ubiquitin recognition factor in ER-associated degradation protein 1	Smp_196510.1	GCGGTACAGGTTATCGGTTAG	ACTTCCAGGTTGATAATTGTAGTTTG	18.8	
Zinc finger SWIM domain-containing protein 3	Smp_035540.1	TGGTGTGTGTACTTGTTCGC	ATGAGATCTAGACCACCGCG	22.2	
Vacuolar protein sorting-associated protein 51 homolog	Smp_340500.2	CGCGAATCATTACTTTTGGGTG	ACGAAATGCCACAGATGAATTTG	21.5	
Uncharacterized aarF domain-containing protein kinase 5	Smp_341650.1	TGTCGTTCTCATGGTGATCC	ACTTGACCTATAGCATATAACAATTG	20.6	
Ubiquitin-protein ligase E3B	Smp_137040.1	GCGTAATTCGATGGTTATGGG	CAAGATTAGCGAACCCCAAAAG	21.4	
Zinc finger and BTB domain-containing protein 17	Smp_347480.1	TGATGGTGACGTTGCTGTAG	TCGACTCTTCCTAATTCTGACGA	22.4	
Transcription factor tau subunit sfc6	Smp_342240.1	GATGCAGAGCTTCAACACTTTC	AAATCCAATTCAGGTTTCTTAGGTAC	24.5	
Ankyrin repeat domain-containing protein 49	Smp_069840.1	CAGCGGGTGCAGATTTAAATG	AATGTCTCAGGTCCTTGGTTG	23.8	
SAP30-binding protein	Smp_013930.1	TTACTAGCACAGACAAACCCAG	ATTCTGAGGATGTGTCATGGG	21.8	
Ribose-phosphate pyrophosphokinase 1	Smp_022090.1	ACATGCCAGTCAAATTCAAGGA	ACATCTCCAACAAGAACCATACG	22.1	
RNA-binding protein 8A	Smp_002550.1	TCCGTAGAAGGTTGGATCTTGT	TAACCAGTTCGCCGATCAAG	20.6	
Nuclear factor related to kappa-B-binding protein	Smp_341400.1	GAAATTGTTCGAGACGCTGTG	TCGATCCAACGCAGAACTAAC	20.2	
Mitochondrial 28S ribosomal protein S14	Smp_090920.1	CACCAGCTCATCATAATAATCCA	TAGCATCCTGAAAGCCACGA	20.2	^20,26,34^
Putative dynactin subunit	Smp_062630.1	GGAATGATGTGGCCGATAGT	CGCAGAGATTGGCTAAATTG	19.2	^20,26^
Eukaryotic translation initiation factor 4e	Smp_001500.1	TGTTCCAACCACGGTCTCG	TCGCCTTCCAATGCTTAGG	20.9	^35,36^
Glyceraldehyde-3-Phosphate dehydrogenase	Smp_056970.1	TGAGGAAATCAAGGCTGCAGT	CCCTTCAATGGTCCAGATGC	17.7	^37–39^
Triosephosphate isomerase	Smp_003990.1	CATACTTGGACATTCTGAGCGTAGA	ACCTTCAGCAAGTGCATGTTGA	20.5	^40,41^
α-Tubulin	Smp_090120.1	CCATTTATGATATTTGTCGACGGA	TTTGTGTAGGTTGGACGCTCTATATCTA	19.3	^41–46^
Cytochrome c oxidase subunit I	Smp_900000.1	TACGGTTGGTGGTGTCACAG	ACGGCCATCACCATACTAGC	13.9	^47–49^
Actin	Smp_307020.1	CGTTGGACGACCTCGACAT	TGTCTTTCTGACCCATACCAACC	16.2	^38,50^

^a^Smp code stands for *Schistosoma mansoni* protein-coding gene ID. Mean Cq stands for Mean Corrected Cq, as described in the Methods. Refs. stands for the previous publications in which the corresponding primer pair of a reference gene was used.

### Expression levels and stability of candidate reference genes measured in RT-qPCR assays

RNAs extracted from six different life-cycle stages of *S. mansoni* (eggs, miracidia, cercariae, 48-h-schistosomula, adult males and adult females), were used in RT-qPCR assays. The RNA integrity of samples in each of the four biological replicates of each *S. mansoni* life-cycle stage was confirmed as being very good, as visualized by their Electropherogram Summary in Supplementary Fig. S2. The amplicon specificities of the 33 candidate reference genes were confirmed by the presence of a single peak in the melting curve analysis obtained at the end of the real-time RT-qPCR assay (Supplementary Fig. S3).

**Figure 3:**
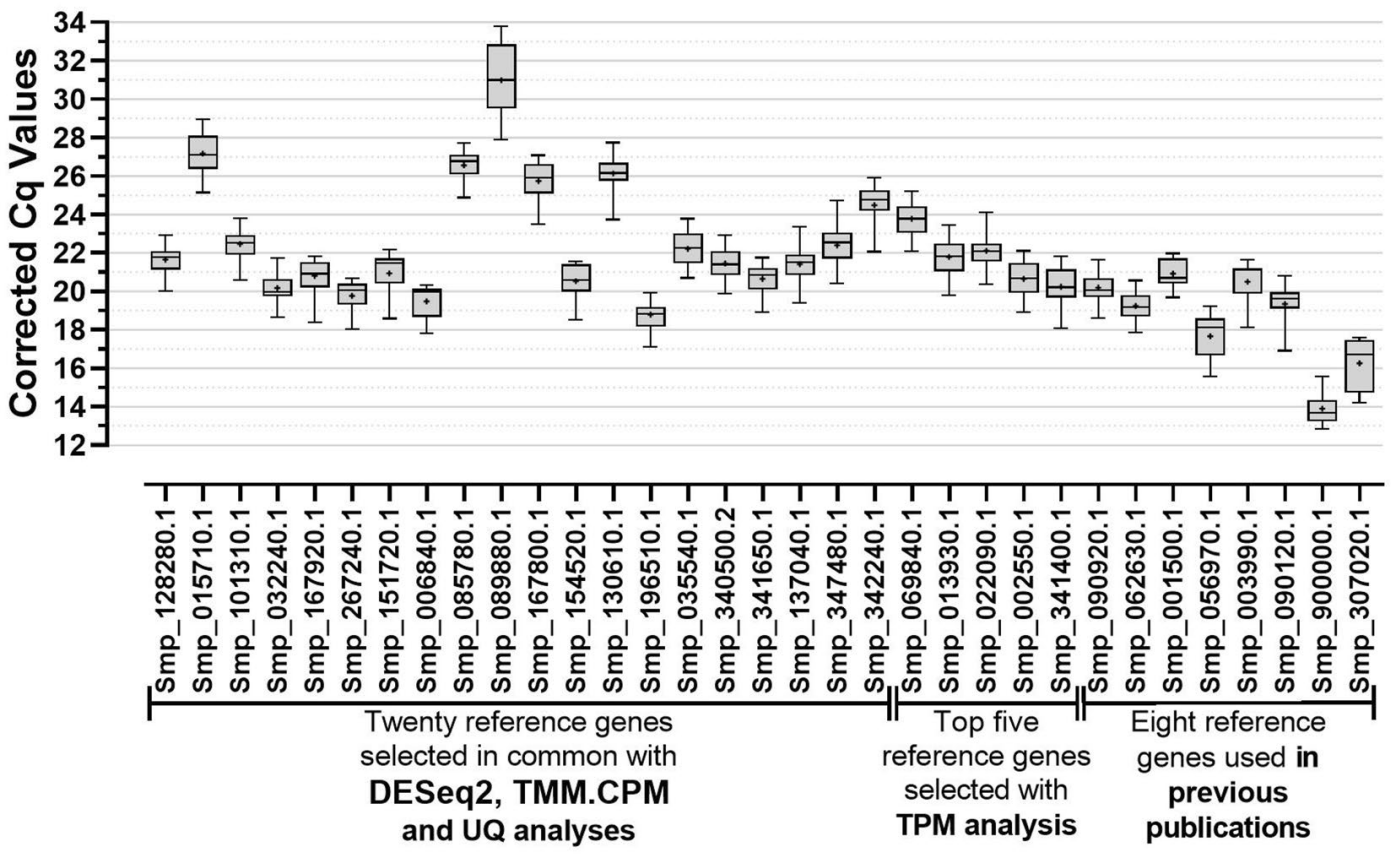
Gene expression analysis performed by RT-qPCR for all 33 candidate reference genes across six different *S. mansoni* life-cycle stages. The data are presented as corrected RT-qPCR quantification cycle (Cq) values as described in the Methods. The Cq values derive from the quantitative RT-qPCR analysis of the genes' expression in the following *S. mansoni* life-cycle stages: eggs, miracidia, cercariae, 48-h-schistosomula, adult males, and adult females. For each gene, the horizontal line represents the median while the + signal represents the average. The boxes and whiskers represent the inter-quantile and min to max ranges, respectively.

The quantification cycle (Cq) values for each candidate reference gene were retrieved from each analyzed sample's real-time PCR amplification curves. Our real-time RT-qPCR gene expression analysis in eggs, miracidia, cercariae, 48-h-schistosomula, adult males, and adult females showed a Cq in the range 13–34 across all candidate reference genes (Fig. 3). The previously reported reference genes *Cytochrome C oxidase subunit I* (Smp_900000.1), *Actin* (Smp_307020.1) and *GAPDH* (Smp_056970.1) exhibited the highest expression levels (lowest Cq values) (Fig. 3). Conversely, the genes with the lowest expression levels (highest Cq values) were *FAD-dependent oxidoreductase domain-containing protein 1* (Smp_089880.1), *6-phosphofructo-2-kinase/fructose-2,6-bisphosphatase 1* (Smp_015710.1), and *39S ribosomal protein L10, mitochondrial* (Smp_085780.1) (Fig. 3 and Supplementary Table S8). Of note, Smp_089880.1 showed a mean corrected Cq value across the six developmental stages higher than 30 (Cq = 31.0) and, therefore, is not recommended to be used as a reference gene.

**Figure 4:**
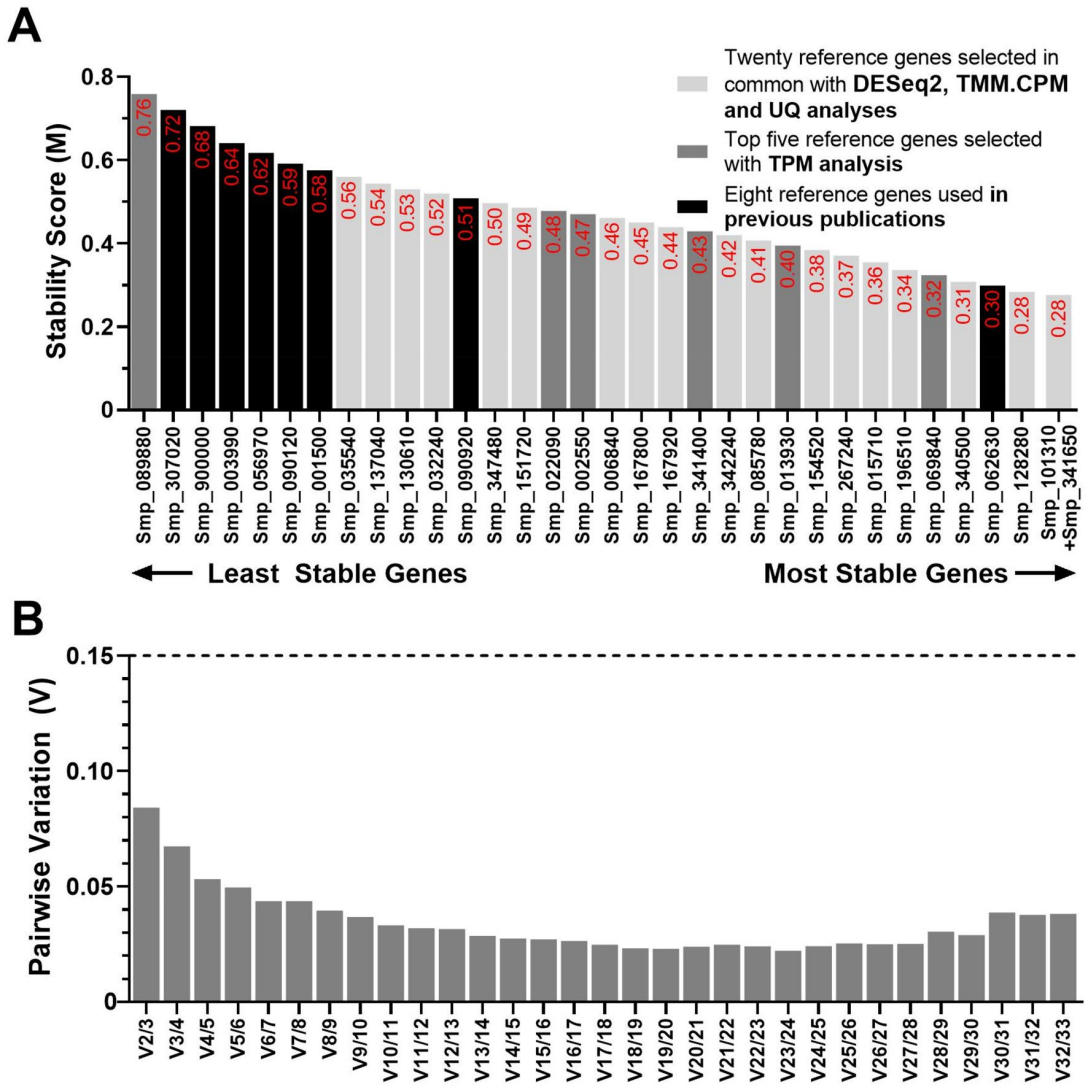
Gene expression stability and ranking of the 33 candidate reference genes across six *S. mansoni* developmental stages measured by RT-qPCR and analyzed with geNorm. (**A**) Expression stability scores (M) of all candidate reference genes are represented at the column bar's top. Genes were colored based on their selection method: genes selected with DESeq2, TMM.CPM and UQ RNA-Seq normalization methods are shown in light grey bars; genes selected with TPM RNA-Seq normalization method are shown in dark grey bars, while genes used in previous works are shown in black bars. The higher the stability score, the least stable the gene. (**B**) Pairwise variation (Vn/n + 1) analysis between the normalization factors NFn and NFn + 1 to determine the optimal number of reference genes required for accurate normalization, where n is the number of genes involved in the normalization factor. The cut-off value determined by this analysis is 0.15.

The Stability Score (M value) of each of the 33 candidate reference genes was calculated using the geNorm software (version 3.5)^21^. This software recommends using a stability score below the threshold of 1.5 to correctly identify reference genes with stable expression. Notably, Smp_341650.1 (M = 0.28), Smp_101310.1 (M = 0.28), and Smp_128280.1 (M = 0.28) were the most stably expressed genes with M values way lower than those from the previously used reference genes (Fig. 4A). The genes with the highest M values (i.e., least stable genes) were Smp_089880.1 (*FAD-dependent oxidoreductase domain-containing protein 1*), Smp_307020.1 (*Actin*), and Smp_900000.1 (*Cytochrome C oxidase subunit I*) (Fig. 4A). Notably, apart from Smp_062630.1, a *Putative dynactin subunit*, all the reference genes used in previous publications were amongst the least stable candidates, as shown by black bars in Fig. 4A. One of the candidate reference genes selected from the TPM normalization method was determined as the least stable candidate (Smp_089880.1). The best scored from this group ranked sixth place (Smp_069840.1, an *Ankyrin repeat domain-containing protein 49*).

Pairwise variation analysis demonstrated that the combination of Smp_101310.1 and Smp_341650.1 was sufficient for accurate gene expression normalization (V2/3 = 0.084) across the six developmental stages (Fig. 4B). Thus, based on geNorm analysis, we conclude that *Histone H4 transcription factor* (Smp_101310.1) and *Uncharacterized aarF domain-containing protein kinase 5* (Smp_341650.1) were the most stable reference genes.

**Figure 5:**
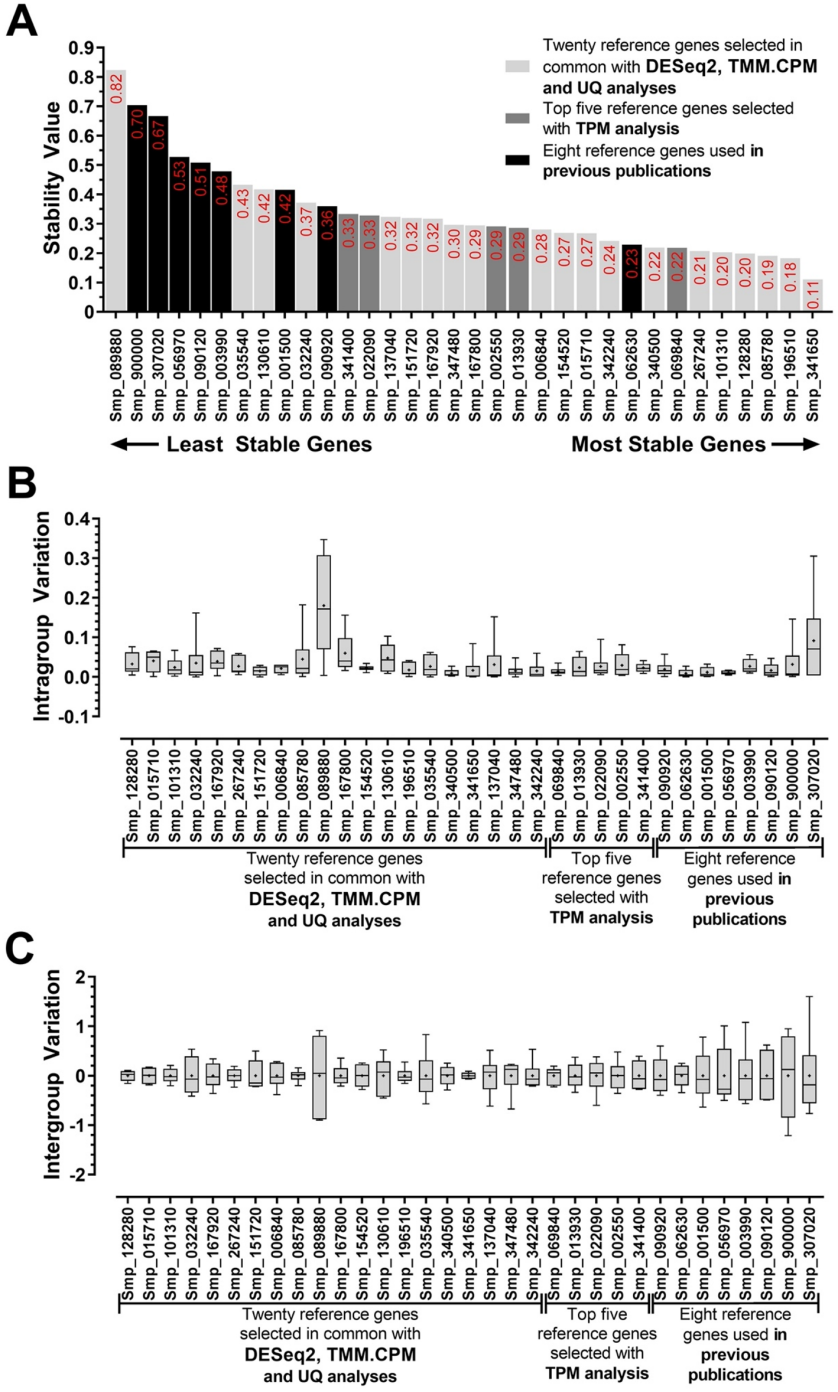
Gene expression stability and ranking of the 33 candidate reference genes across six *S. mansoni* developmental stages measured by RT-qPCR and analyzed with NormFinder. (**A**) The stability value of all candidate reference genes is represented at the column bar's top. Genes were colored based on their selection method: genes selected with DESeq2, TMM.CPM and UQ RNA-Seq normalization methods are shown in light grey bars; genes selected with TPM RNA-Seq normalization method are shown in dark grey bars, while genes used in previous works are shown in black bars. The higher the stability score, the least stable the gene. (**B**) Intragroup variation represents how much the biological replicates varied from each other in each of the six developmental stages. (**C**) Intergroup variation represents how much the average of the biological replicates of each stage varied among the six developmental stages. For each gene, the horizontal line represents the median while the + signal represents the average. The boxes and whiskers represent the inter-quantile and min to max ranges, respectively.

We used a second software, namely NormFinder^51^, to evaluate the most stable candidate reference gene. Notably, the six best reference genes determined by this analysis were also predicted as the most stable genes by our analysis with DESeq2, TMM.CPM and UQ normalization methods (Fig. 5A). The three most stable reference genes were *Uncharacterized aarF domain-containing protein kinase 5* (Smp_341650.1, stability value = 0.11), *Ubiquitin recognition factor in ER-associated degradation protein 1* (Smp_196510.1, stability value = 0.18) and *39S ribosomal protein L10, mitochondrial* (Smp_085780.1, stability value = 0.19). Notwithstanding, as for the geNorm analysis, the least stable reference genes defined with NormFinder were *FAD-dependent oxidoreductase domain-containing protein 1* followed by the *Cytochrome C Oxidase subunit 1*, *Actin*, *GAPDH,* and *Tubulin* (Smp_089880.1, Smp_900000.1, Smp_307020.1, Smp_056970.1, and Smp_090120.1, respectively) (Fig. 5A). The most stable candidate reference gene determined with the TPM analysis was Smp_069840.1, an *Ankyrin repeat domain-containing protein* 49, that scored seventh with the NormFinder software.

NormFinder also allows determining the Intra- and Intergroup variation, defined respectively as the variation among the sample biological replicates within each analyzed stage, and the variation among the stages of the biological replicates average of each different stage analyzed. Conspicuously, intragroup variation was less dispersed than intergroup variation for all candidate reference genes analyzed, but Smp_307020.1 and Smp_089880.1 stood out when it comes to intragroup variation, having a more dispersed expression within the biological replicates of the cercariae and schistosomula groups (Fig. 5B). On the other hand, intergroup variation with all normalization methods was small for almost all candidate reference genes selected by our analysis, except Smp_089880.1 (Fig. 5C). Simultaneously, the genes from previous works, *eIF4e*, *GAPDH, TPI, Tubulin, Cytochrome C oxidase subunit 1* and *Actin* (Smp_001500.1, Smp_056970.1, Smp_003990.1, Smp_090120.1, Smp_900000.1, and Smp_307020.1, respectively) were more dispersed than the other genes. The intergroup variation agreed with our ranking pointed in Fig. 2.

**Figure 6:**
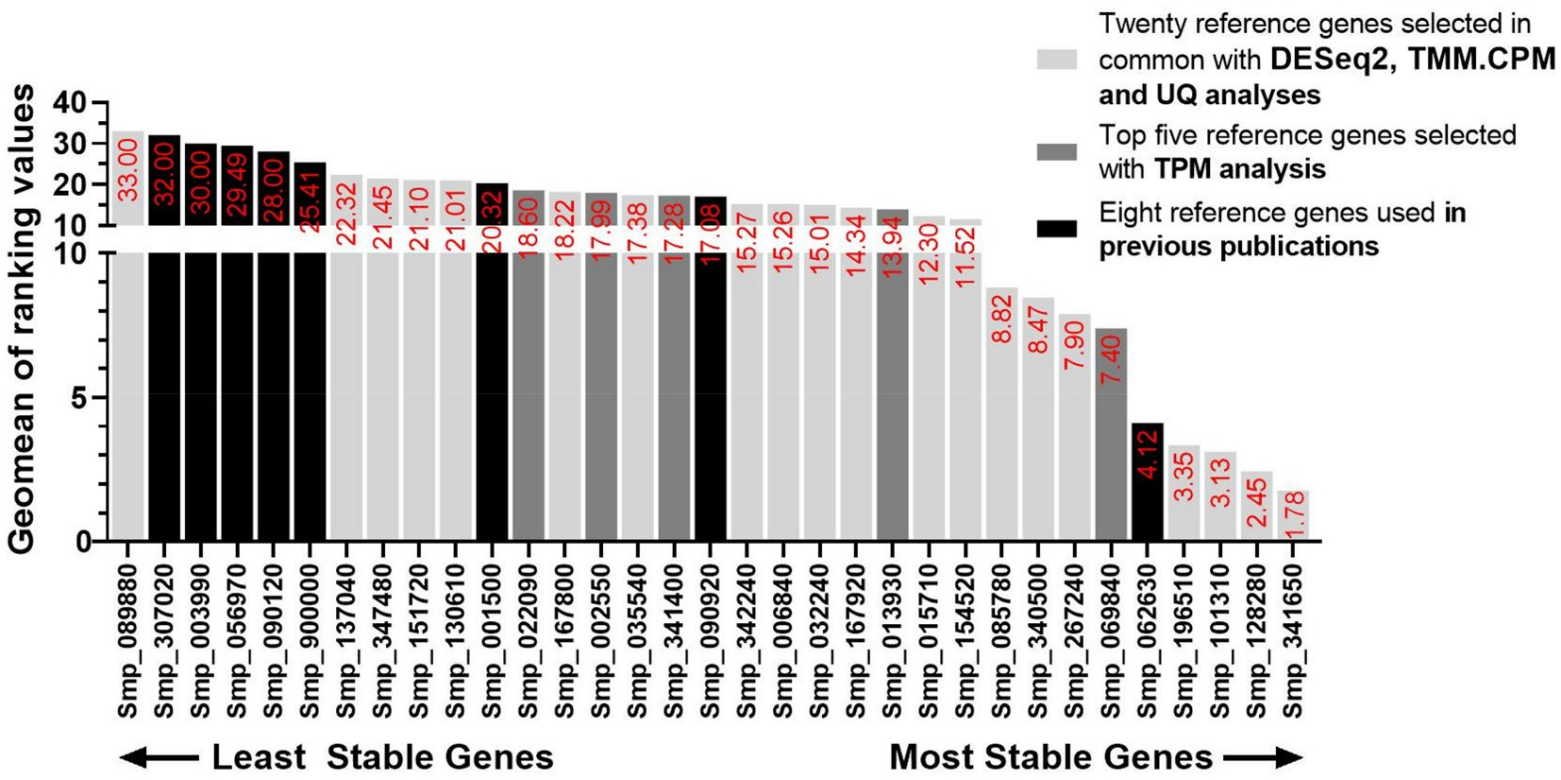
RefFinder comprehensive analysis with corrected Cp values retrieved from RT-qPCR of *S. mansoni* life-cycle stages samples. The geometric mean of all candidate reference genes' stability ranking values calculated by RefFinder is represented at the top of the column bar. Genes were colored based on their selection method: genes selected with DESeq2, TMM.CPM and UQ RNA-Seq normalization methods are shown in light gray bars, genes selected with TPM RNA-Seq normalization method are shown in dark gray bars, while genes used in previous works are shown in black bars. The higher the stability ranking value, the least stable the gene.

We used a third software, namely RefFinder^52^, to identify the most stable reference gene. RefFinder uses Cq values from all samples and genes analyzed as input data and performs a group measurement from all available methods to determine the most to the least stable reference gene. As RefFinder does not take into consideration the primer efficiency for each candidate reference gene tested, we performed this analysis using the corrected Cq value (using the formula = ${\text{Log}}_{2}{{\text{E}}}^{\text{Cp value}}$). RefFinder provides a ranking order for all the candidate reference genes for each of the four methods tested: DeltaCT, BestKeeper, NormFinder, and geNorm (Supplementary Table S9). In the end, the analysis also provides a comprehensive ranking value for each gene that takes into consideration the ranking for each of the four methods tested (Fig. 6). As expected, the ranking obtained by RefFinder was similar to our previous individual analyses with geNorm and NormFinder, pointing as the four most stable genes the ones that were selected based on the DESeq2, TMM.CPM and UQ RNA-Seq normalization methods (Smp_341650.1, Smp_128280.1, Smp_101310.1 and Smp_106510.1) (Fig. 6). In fifth place was Smp_062630.1, a *Putative dynactin subunit* previously used by us^20,26^; the *Ankyrin repeat domain-containing protein 49* (Smp_069840.1) scored sixth place (Fig. 6). The least stable candidate reference was *FAD-dependent oxidoreductase domain-containing protein 1* followed by *Actin*, *TPI*, *GAPDH*, *Tubulin,* and *Cytochrome C Oxidase subunit 1* (Fig. 6)*,* which was all in agreement with the previously tested methods. This agreement could be confirmed when a Pearson correlation was calculated with the results from each of the three tested software (geNorm, NormFinder and RefFinder), one by one, with Pearson correlation coefficient values (r) above 0.93 (a positive linear correlation) and p-values < 0.001 for all cases (Supplementary Fig. S4).

Works in the literature recommend the selection of reference genes that possess different expression levels and biological functions^21,51,53^. This would reduce incorrect normalization eventually due to the reference genes being affected by any treatment to the samples. Furthermore, we have considered that genes without a predicted function or with putative annotation should be avoided to favor best-characterized genes. Unquestionably, Smp_341650.1 has consistently ranked as the best candidate in three of the tested methods. However, it is annotated as an *Uncharacterized aarF domain-containing protein kinase 5*. This protein's function is not yet clear, and no information of its protein kinase activity and what type of substrate it would phosphorylate has been provided until now. The second, consistently most stable reference gene was the *Histone H4 transcription factor* (Smp_101310.1), which was singled out here as the first recommended reference gene, followed by *Ubiquitin recognition factor in ER-associated degradation protein 1* (Smp_196510.1), due to their biological functions and expression levels across all samples. Therefore, our work pointed to Smp_101310.1 and Smp_196510.1 as the reference genes to be used in RT-qPCR experiments with the six tested *S. mansoni* developmental stages.

### Differential gene expression across six developmental stages using the reference genes identified in the present work

**Figure 7:**
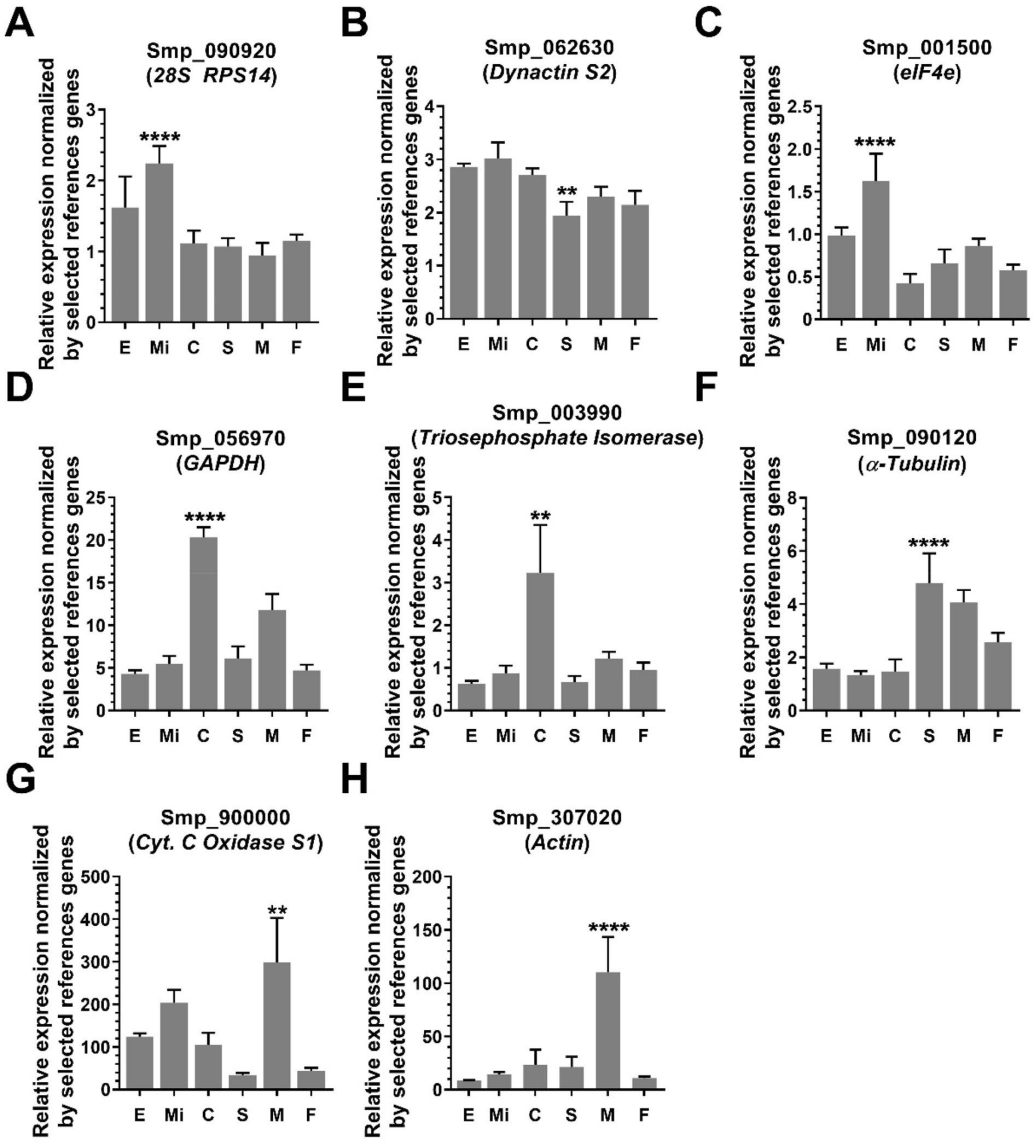
Relative expression at six different *S. mansoni* developmental stages of reference genes previously used in the literature, normalized by the two most stable reference genes found in the present work. (**A**) *26s Ribosomal Protein Subunit 14*; (**B**) *Dynactin subunit 2*; (**C**) *Eukaryotic translation initiation factor 4e*; (**D**) *Glyceraldehyde-3-Phosphate dehydrogenase*; (**E**) *Triosephosphate isomerase*; (**F**) *α-Tubulin*; (**G**) *Cytochrome C oxidase subunit* 1 and (**H**) *Actin*. Quantitative RT-qPCR was performed for the eight genes at six different parasite life-cycle stages, namely egg (E), miracidium (Mi), cercaria (C), 48-h-schistosomulum (S), adult male (M) and adult female (F) (x-axis). The expression values are represented as the relative expression using as normalizer the geometric mean of the two reference genes selected in this work, namely Smp_101310.1 and Smp_196510.1. Bars represent the standard deviation of the mean from four biological replicates for each stage. Three technical replicates were assayed for each of the four biological replicates. One-way ANOVA test was used to calculate the statistical significance of the expression differences among the parasite life-cycle stages (*p-value ≤ 0.05; **p-value ≤ 0.01; ***p-value ≤ 0.001; ****p-value ≤ 0.0001). For clarity purposes, we show only the highest p-value for the stage in which the Smp was detected with the highest expression difference among the life-cycle stages.

We evaluated the differential gene expression levels across the six different developmental stages of the eight reference genes previously used in the literature, using for normalization the geometric mean of the two reference genes recommended here: *Histone H4 transcription factor* (Smp_101310.1) and *Ubiquitin recognition factor in ER-associated degradation protein 1* (Smp_196510.1). All genes previously used in the literature as reference showed a statistically significant differential expression in at least one *S. mansoni* developmental stage tested (Fig. 7). Smp_062630.1, *Dynactin subunit 2* presented the lowest expression level fold-change (1.6-fold, expression level in miracidium compared with schistosomula, Fig. 7B). In contrast, the highest expression level fold-change (11-fold) was observed for Smp_307020.1, *Actin* (expression levels in males compared with eggs, Fig. 7H).

### Evaluation of the performance of the reference genes identified in the present work under different conditions: female pairing status or gene silencing

**Figure 8:**
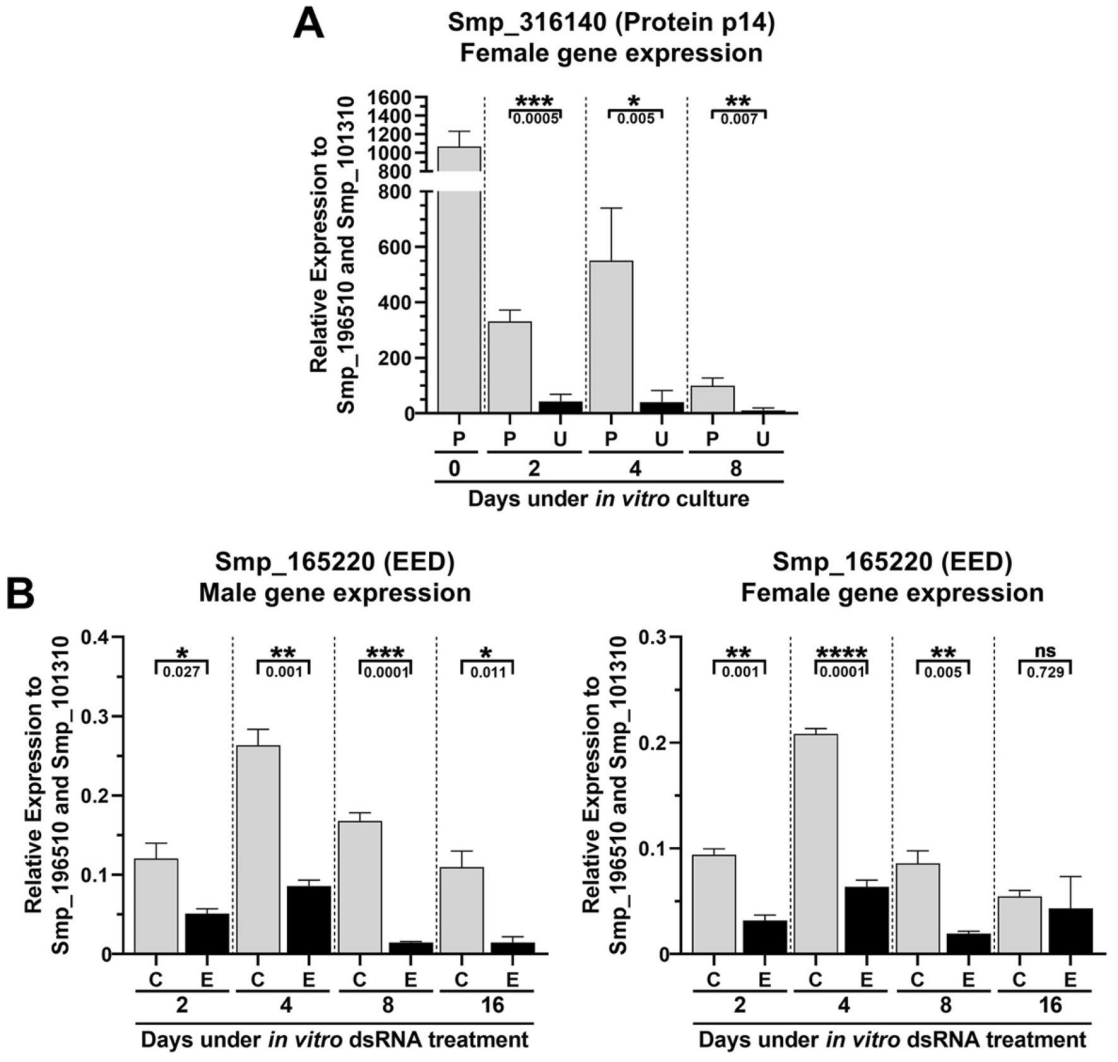
Relative expression of two protein coding genes in *S. mansoni* under different culturing conditions, normalized by the two most stable reference genes found in the present work. (**A**) Female adult worm gene expression pattern of Smp_316140 (Protein p14) across different in vitro culturing conditions. Quantitative RT-qPCR was performed with RNA samples from females that were paired (P) or unpaired (U) to males and cultivated in vitro for 2, 4 or 8 days. Day 0 stands for paired females retrieved right after perfusion. (**B**) Male and female adult worm Smp_165220 (EED) gene expression in samples obtained from couples treated with dsRNA targeting either the control unrelated dsmCherry gene (C) or the Smp_165220 (EED) gene (E) in vitro for 2, 4, 8 or 16 days. The expression values are represented as the relative expression using as normalizer the geometric mean of the two reference genes selected in this work, namely Smp_101310.1 and Smp_196510.1. Bars represent the standard deviation of the mean from three biological replicates for each experiment. Three technical replicates were assayed for each of the three biological replicates. Unpaired student t-test was used to calculate the statistical significance of the expression differences in the comparisons (*p-value ≤ 0.05; **p-value ≤ 0.01; ***p-value ≤ 0.001; ****p-value ≤ 0.0001; ns = p-value > 0.05). The p-value obtained from the Student’s t-test is represented under the brackets.

We evaluated under two other different conditions the performance of the two most stable reference genes found in this work. The first condition involved adult females cultured in vitro for 8 days as unpaired worms or paired with males. The stability of the two normalizer genes identified here was compared with the stability of *Actin*, *GAPDH*, *Tubulin*, as well as of *LETM1* (Smp_065110) and *PSMB7* (Smp_073410), two genes identified as normalizers to study gene expression in *S. mansoni* males from single-sex and dual-sex infections^25^. RT-qPCR data were obtained for these genes from a total of 21 samples covering three biological replicates of females cultured for 0, 2, 4 and 8 days either paired with males or as separated female worms. Gene stability was calculated from the RT-qPCR data, which was analyzed with the three algorithms, geNorm, NormFinder and RefFinder (Supplementary Table S10); the analysis showed that *Ubiquitin recognition factor in ER-associated degradation protein 1* (Smp_196510.1) was identified by the three algorithms as the most stable normalizer gene, whereas *Histone H4 transcription factor* (Smp_101310.1) was identified as the second most stable normalizer by geNorm and RefFinder (Supplementary Table S10). We measured in females the pairing-dependent expression of the *eggshell protein p14* (Smp_316140.1)^54,55^ using the two reference genes identified here (Fig. 8). Expression of *p14* was significantly reduced ~ eightfold along the eight days in culture in unpaired females compared with paired ones (Fig. 8A). A similar expression pattern was obtained when using *Actin*, *GAPDH* and *Tubulin* as normalizers (Fig. S5A).

The second condition involved adult worm pairs treated in culture for 16 days with double-stranded RNAs that targeted and silenced the protein-coding gene *EED* (dsEED) (Smp_165220, *Embryonic Ectoderm Development*), a component of the Polycomb Repressive Complex 2 (PRC2), which is the histone modifying complex that tri-methylates H3K27. A non-related double-stranded RNA targeting the mCherry gene (dsmCherry) was used as a negative control. Again, the stability of the two reference genes identified here was compared with the stability of three reference genes from the literature, namely *Actin*, *GAPDH* and *Tubulin*, using RT-qPCR data obtained from 48 samples corresponding to three biological replicates of males and females exposed in vitro to dsEED or dsmCherry along 2, 4, 8 and 16 days in culture. The stability analysis showed that *Tubulin* (Smp_090120.1) was the most stable gene (Supplementary Table S11), whereas *Ubiquitin recognition factor in ER-associated degradation protein 1* (Smp_196510.1) and *Histone H4 transcription factor* (Smp_101310.1) were identified as the second and third most stable normalizers (Supplementary Table S11). Using the latter two normalizer genes, a significant knockdown of *EED* was detected by RT-qPCR upon the exposure of adult males and females in culture for 2, 4 and 8 days to dsRNAs targeting the *EED* gene compared with a control dsRNA (Fig. 8B). A similar expression pattern was obtained when using *Actin*, *GAPDH* and *Tubulin* as normalizers (Fig. S5B).

## Discussion

A decade ago, the use of high throughput transcriptomic technologies, such as RNA-Seq, was restricted due to its high price and to the limited number of powerful informatics tools for proper analysis. Nowadays, high throughput expression analyses are usually just the starting point of major studies, providing us with a plethora of processed data. While large amounts of data have been explored, further steps to provide the functional characterization of candidate genes and gene families have to be performed gene by gene. The first step in this direction is to confirm gene expression patterns individually, and real time quantitative RT-PCR has been one of the most used techniques.

The raw read counts data from RNA-Seq experiments need to be converted into informative measurements of gene expression. Normalization is a crucial step towards resolving the variabilities that affect the number of reads mapped to a gene, such as the gene length^56^, GC-content^57^ and the sequencing depth^58^. These RNA-Seq library features have to be considered when performing comparisons between different samples, and the major source of variation between RNA-Seq data is the sequencing depth that is reflected by the library size (i.e., how many reads are generated from the experiment). Library size normalization relies on scaling raw read counts in each sample by a single sample-specific factor reflecting its library size. The most commonly used methods include Relative Log Expression that is the basis of DESeq2 analysis, Upper Quartile (UQ) and Trimmed Mean of M-values (TMM), and all these methods have been thoroughly reviewed^59–64^. Another frequently used RNA-Seq normalization method is TPM, which relies on both the gene length and the sequencing read length corrections. However, TPM has been pointed as a flawed normalization method for RNA-Seq normalization between highly different samples^56,65^, and could be less appropriate for the available *S. mansoni* data at different life-cycle stages. Some recent evidence points to the misuse of TPM for normalization of RNA-Seq data^61,66^. Our RT-qPCR results show that candidate reference genes from the TPM normalization method have been detected as the least stable reference genes for the correct normalization in samples from the six S. *mansoni* life-cycle stages tested.

We used three different and well-established RT-qPCR analysis tools that follow distinct strategies to determine the most stable reference genes. It has already been shown that the golden standard for selecting reference genes relies on using these three different approaches^67–70^. GeNorm^21^ is based on paired dependent gene comparison. The expression level of each candidate reference gene is compared with the normalization factor, excluding the least stable genes one by one until the two genes with the highest stability (least variation) remain. NormFinder^51^, on the other hand, determines intragroup and intergroup variation between the different sample groups to identify the genes with the highest expression stability. RefFinder^52^ is a tool that gathers four computational programs (geNorm, NormFinder, BestKeeper and the comparative Delta-Ct method) to compare and rank the tested candidate reference genes. Although it does not use primer efficiency for the ranking, this limitation can be overcome by using corrected Cq values.

We have defined Smp_101310.1 and Smp_196510.1 as the most stable reference genes in our expression stability analysis of the six different *S. mansoni* life-cycle stages derived from the RT-qPCR data. Smp_101310.1 (*Histone H4 transcription factor*) has been computationally annotated as a transcription factor that activates Histone H4 gene expression. In humans, the gene *Histone H4 Transcription Factor*/*HINFP* is categorized as having “Low tissue and cell specificities”, is detected in all tissues and is expressed at similar levels in almost all tissues. Human HINFP is a critical component of a signaling pathway that controls expression of histone H4 genes^71^. Human HINFP has been shown to possess a HINFP-specific conserved region that is present in HINFP homologues of all metazoan species that have been examined^72^. Therefore, it is likely that in *S. mansoni*, Smp_101310 gene expression pattern is similar to that of human HINFP, with low differential expression along the tissues and stages of the parasite, which would be necessary to maintain the expression of histone H4 target genes.

Smp_196510.1 was computationally annotated as the *Ubiquitin recognition factor in ER-associated degradation protein 1*, an essential component of the ubiquitin-dependent proteolytic pathway that degrades ubiquitin fusion proteins. In humans, the gene *Ubiquitin Recognition Factor In ER Associated Degradation 1*/*UFD1* is categorized as having “Low tissue and cell specificities”, is detected in all tissues and is expressed at similar levels in almost all tissues. In humans, Ufd1 facilitates the exportation of misfolded proteins from the endoplasmic reticulum to the cytosol for ubiquitin–proteasome pathway (UPP) degradation^73^. This regulated protein degradation by the ubiquitin–proteasome system plays a central role in diverse cellular processes. In *S. manson*i, it has been already shown that functional UPP components are required for parasite development in the vertebrate host^74^. In fact, siRNA knockdown of the SmRPN11/POH1 proteasome subunit gene decreased schistosomula viability by 78%, suggesting an important role for UPP in *S. mansoni* development and survival^75^. Therefore, some of the genes that participate in the UPP, such as Smp_196510, are likely to be highly controlled and expressed in stable levels throughout the life-cycle stages of the parasite. On the other hand, an interesting observation arising from our work is that *GAPDH*^76^, a key enzyme of glycolysis, *actin*^77^, a cytoskeleton component, and *TPI* (*triosephosphate isomerase*)^78^, another glycolysis component enzyme, were the least stable genes in our analysis. Human *GAPDH* gene is enriched in skeletal muscle and shows higher tissue and cell specificities. In *S. mansoni*, while *SmGAPDH* is found within all schistosome tissues, the protein has also been particularly identified in the parasite tegument^79–81^, which is an abundant tissue in adult worms when compared with other life-cycle stages.

Smp_101310.1 and Smp_196510.1 have average Cq values of 22 and 19, respectively, across the six developmental life-cycle stages tested. It is recommended that reference genes should have expression levels in the same range as the genes of interest being tested. Therefore, if genes of interest that are being quantified across life-cycle stages are lowly expressed, such as long non-coding RNA genes^82,83^, we recommend the inclusion of a third reference gene Smp_085780.1 (*39S ribosomal mitochondrial protein L10*), a mitochondrial large ribosomal subunit component gene that was detected with an average Cq of 26 in the RT-qPCR assays.

By using the geometric mean of Smp_101310.1 and Smp_196510.1, the two reference genes recommended here, for the expression normalization of the genes commonly used as reference in previous works, we found that Smp_090920.1 (*28s ribosomal protein subunit 14*), Smp_062630.1 (*Dynactin subunit* 2), Smp_001500.1 (*eIF4e*), Smp_056970.1 (*GAPDH*), Smp_003990.1 (*Triosephosphate isomerase*), Smp_090120.1 (*α-tubulin*), Smp_900000.1 (*Cytochrome C oxidase Subunit 1*) and Smp_307020.1 (*Actin*) were differentially expressed in at least one *S. mansoni* developmental stage*,* and therefore should not be used for RT-qPCR normalization purposes when analyzing different parasite stages. It is also worth mentioning that whereas our analysis comprised six different developmental stages, this might not be the case for all the previous works in the literature mentioned here, and therefore the reference genes used in some of them may have been adequate for those specific works.

Expression stability analysis indicated that Smp_101310.1 and Smp_196510.1 were also suitable for RT-qPCR data normalization in experiments involving the pairing status of females in culture, or the exposure of parasites to dsRNAs in gene-silencing assays. Therefore, while it is recommended to define stable reference genes in the condition that is being specifically tested, the genes pointed here can be used as starting points when selecting reference genes for correct normalization under such similar experimental conditions.

In conclusion, this work is the first systematic study that screened and validated the optimal reference genes for RT-qPCR relative gene quantification in six *S. mansoni* life-cycle stages. Twenty-five novel candidate reference genes and eight previously used candidate reference genes were selected, and their expression stabilities evaluated. We recommend as the reference genes of choice two genes, Smp_101310.1 and Smp_196510.1, which were the most stably expressed at the six different *S. mansoni* developmental stages that were analyzed.

## Methods

### RNA-Seq analyses

The selected RNA-Seq libraries were downloaded from the SRA-NCBI database using the SRA Toolkit^84^ v.2.10.8. Adapters and bad quality reads were filtered out using fastp v. 0.20.0 with default parameters^85^. Filtered reads were mapped against the *S. mansoni* genome v.7 using STAR^86^ v. 2.7.0f, and transcripts were quantified with RSEM^33^ v1.3.0 (estimate-rspd parameter on). The GTF file (available at http://schistosoma.usp.br/) containing the protein-coding transcriptome v 7.1 (WBPS14)^19^ merged with the lncRNAs transcriptome^20^ identified by Maciel et al. was used as a reference during the quantification process. As suggested by DESeq2^30^, after reads counting with RSEM^33^ we performed a minimal pre-filtering to keep only genes that had at least 10 reads total when adding all stages. This resulted in 13,624 protein-coding genes (out of 14,548 genes in the v 7.1 transcriptome^19^) and in 9391 lncRNAs (out of 16,583 lncRNAs^20^) that were considered for further analyses.

Counts and TPM values obtained with RSEM were imported to R^87^ environment v. 3.6.3 with the tximport package^88^ v. 1.14.2. Normalized counts were obtained with DESeq2^30^ v. 1.24.0, edgeR^31^ v. 3.28.1 (calcNormFactors with TMM method and exported in CPM) and EBSeq^32^ v. 1.2.6 (QuantileNorm with quantile = 0.75) packages. The Coefficient of Variance (CV) for each transcript in each normalization method was calculated as the ratio of the standard deviation (SD) σ to the arithmetical mean μ.

A user-friendly web application, built with shiny R^89^, showing the expression along the stages for each of the normalization methods and their respective CV values is available at https://verjolab.shinyapps.io/Reference-genes/.

### Ethics statement

The experimental protocols were in accordance with the Ethical Principles in Animal Research adopted by the Conselho Nacional de Controle da Experimentação Animal (CONCEA) and the protocol/experiments have been approved by the Comissão de Ética no Uso de Animais do Instituto Butantan (CEUAIB number 8859090919). This study was carried out in compliance with the ARRIVE guidelines (http://www.nc3rs.org.uk/page.asp?id=1357).

### Parasite material

The BH strain (Belo Horizonte, Brazil) of *S. mansoni* was maintained in the intermediate snail host *Biomphalaria glabrata* and as definitive host the golden hamster (*Mesocricetus auratus*). Female hamsters aged 4 weeks, freshly weaned, weighing 50–60 g, were housed in cages (30 × 20 × 13 cm) containing a sterile bed of wood shavings. A standard diet (Nuvilab CR-1 Irradiada, Quimtia S/A, Paraná, Brazil) and water were made available ad libitum. The room temperature was kept at 22 ± 2 °C, and a 12:12 h light–dark cycle was maintained.

Hamsters were infected with an *S. mansoni* cercariae suspension containing approximately 200–250 cercariae via subcutaneous injection^90^. After 49 days of infection, *S. manson*i adult worms were recovered by perfusion of the hepatic portal system^91^. *S. mansoni* eggs were extracted from *S. mansoni* infected female hamsters livers, and miracidia were hatched from *S. mansoni* eggs, both as previously described^92^. Cercariae were harvested from infected *B. glabrata* snails exposed to light and mechanically transformed to obtain schistosomula in vitro^93^. The newly transformed schistosomula were maintained for 48 h in M169 medium (Vitrocell, cat number 00464) supplemented with penicillin/streptomycin, amphotericin, gentamicin (Vitrocell, cat number 00148), 2% fetal bovine serum, 1 μM serotonin, 0.5 μM hypoxanthine, 1 μM hydrocortisone, and 0.2 μM triiodothyronine at 37 °C and 5% CO2 before collection and RNA extraction.

### Adult worm pairing and EED silencing assays

Adult worm pairing and silencing experiments were performed in ABC media as previously reported^94^. At day 42 after infection, *S. mansoni* adult worms were recovered by perfusion of the hamster hepatic portal system. For the pairing experiments, ten adult worm paired couples, or unpaired females naturally recovered from the hamster perfusion were cultivated in 6-well plates containing 5 mL of ABC media for 2, 4, or 8 days and 70% of the media was exchanged every other day. For the silencing experiments, adult worm paired couples were treated with 30 µg/mL/day of dsRNA targeting either Smp_165220 (EED) or a control unrelated gene (mCherry) in 6-well plates containing 5 mL of ABC media for 2, 4, 8 or 16 days.

At the end of the experiment, the adult worm couples were collected and stored in *RNAlater* (Thermo Fisher) for further manual separation of males and females before RNA extraction.

### dsRNA synthesis

Double-stranded RNA (dsRNA) was synthesized from DNA templates amplified from cDNA of male and female adult worms, using the specific primer sequences indicated below, all of them containing in addition a 17-nt T7 RNA Polymerase Promoter sequence at the 5′-end. For Smp_165220.1 (EED) silencing, three different regions of the transcript were targeted, with three different dsRNAs that were obtained from the in vitro transcription of the three amplicons generated: Region 1—Primer F 5′–ACAGATAGTTCTGTGCAGACTCAA–3′ and Primer R 5′–AGCGGATCAGTTGGTTGACTT–3′; Region 2—Primer F 5′–CCTGTGCTTGTTCAACACTTCC–3′ and Primer R 5′–GGACCAACTCCACTAACTGTAGG–3′ Region 3—Primer F 5′–TGTAGTCTGAAGAATGATCTGGAAGA–3′ and Primer R 5′–CGATCCGTGACCAACAAGACTA–3′. The in vitro dsRNA transcription reaction was adapted from a tRNA transcription protocol^95^. Briefly, reactions were performed at 37 °C for 12 h in a buffer containing 40 mM Tris–HCl (pH 8.0), 22 mM MgCl_2_, 5 mM DTT, 2 mM spermidine, 0.05% BSA, 15 mM guanosine monophosphate, 7.5 mM of each nucleoside triphosphate, amplified template DNA (0.1 µg/µL) and 5 µM of T7 RNA polymerase. The transcribed dsRNA was treated with DNase at 37 °C for 30 min and precipitated using 1:10 (v/v) 3 M sodium acetate pH 5.2 and 1:1 (v/v) of isopropanol. The pellet was washed twice with 70% ethanol and then eluted in water to reach a final concentration of 3 µg/µL. Double-stranded RNA (30 µg/mL/day) was provided to the parasites via soaking. The mCherry gene was used as a non-related dsRNA control and was amplified from a pPLOT-mCherry plasmid containing the mCherry gene with the following primers, containing in addition a T7 RNA Polymerase Promoter sequence at the 5′-end: Primer F 5′–TGGAAGGTTCTGTAAATGGACA–3′ and Primer R 5′–CTCCCTCAGCCCTTTCGTAT–3′.

### RNA extraction and cDNA synthesis

Total RNA from the eggs, miracidia, cercariae, schistosomula, adult males and adult females was extracted using the Qiagen RNeasy Plus Micro Kit (Cat number 74034). Briefly, 55,000–70,000 eggs, 30,000–45,000 miracidia, 40,000–55,000 cercariae, 40,000–65,000 schistosomula, 40 adult paired males and adult paired females for each of four biological replicates were grounded with glass beads in buffer RLT supplemented with 2-mercaptoethanol, according to Qiagen recommendation, for 2 min and then frozen in liquid nitrogen. After freezing, the samples from eggs, cercariae, and schistosomula were thawed in a heat block at 65 °C and submitted to a grinding, freezing, and de-freezing process twice more. Miracidia, male and female adult worms were ground only once and frozen and thawed three times. The protocol was followed according to the manufacturer's instructions, including gDNA exclusion by the provided gDNA elimination column. All RNA samples were quantified using the Qubit RNA HS Assay Kit (Q32852, Thermo Fisher Scientific), and the integrity of RNAs was verified using the Agilent RNA 6000 Pico Kit (5067–1513, Agilent Technologies) in a 2100 Bioanalyzer Instrument (Agilent Technologies). For quantitative RT-qPCR analysis across the different life cycle stages of *S. mansoni*, complementary DNAs were obtained by reverse transcription (RT) of 400 ng total RNA using SuperScript IV Reverse Transcriptase (18091050, Invitrogen) and random hexamer primers in a 20 μL volume, according to the manufacturer's recommendations. For the pairing status experiments, cDNA was synthetized from 1000 ng of total RNA and for the silencing experiments, cDNA was obtained from 200 ng of total RNA.

### Quantitative RT-qPCR assays and analyses

Thirty-three candidate reference genes were selected for RT-qPCR assays and analysis in this study. Twenty-five candidate reference genes were selected based on a re-analysis of published data, and eight candidate reference genes were selected because of their frequent use in previous publications (Table 1 and Supplementary Table S6). All primer pairs were designed using PrimerQuest Tool provided by IDT Integrated DNA Technologies (https://www.idtdna.com/PrimerQuest/) with PCR amplicon length ranging from 50 to 300 bp and melting temperature (Tm) of approximately 60 °C. All primer sequences are reported in Table 1 and the predicted amplicon sizes and primers efficiencies are shown in Supplementary Table S7. After reverse transcription, the obtained complementary DNAs (cDNAs) were diluted eight times in water. Quantitative PCR was performed using 2.5 µL of each diluted cDNA in a total volume of 10 μL containing 1× LightCycler 480 SYBR Green I Master Mix (04707516001, Roche Diagnostics), 800 nM of each primer in a LightCycler 480 System (Roche Diagnostics), and each real-time qPCR was run in three technical replicates. The PCR conditions included initial activation at 95 °C for 10 min; 45 cycles with denaturation at 95 °C for 10 s, annealing at 60 °C for 10 s and extension at 72 °C for 20 s. A dissociation step (95 °C for 15 s, 60 °C for 1 min, 95 °C for 15 s, 60 °C for 15 s) was added at the end of the run to confirm the amplicon specificity for each gene. The quantitative RT-qPCR assays were performed following the MIQE guidelines^21,51,53^.

The amplification efficiency for each primer was calculated using a diluted series of cDNA synthesized using five µg of RNA from *S. mansoni* male and female adult worms. The cDNAs were mixed and diluted in a 3× factor starting from 1:8 dilution. The average Cq values from all three technical replicates in at least four different dilutions were used to retrieve a linear regression. The x-axis corresponds to the log_10_ dilution factor. The slope from the linear regression curve was used to get the primer efficiency value (**E**), where $\mathbf{E}= {10 }^\frac{1}{Slope}$. The **E** value is then used in the equation where Primer Efficiency (%) = ((**E** − 1)*100). An **E** value of 2 equals to a Primer Efficiency of 100%; therefore, the slope needs to be around 3.32.

Three different tools were used to evaluate gene expression stability using RT-qPCR data. The first one is geNorm^21^, which requires the transformation of quantification cycle (Cq) values to relative quantities, ranging from 0 to 1. The mean Cq values from the triplicate runs were used as input data and converted into relative quantities using the lowest Cq values mean of each primer as reference for normalization. It is important to note that the primer efficiency value **(E)** is used to retrieve the relative quantities by using the equation $\text{Relative quantity}= \, {\text{E}}^{\text{Lowest Cp value}-\text{Cp value}}$. This program estimates an expression stability value (M) for each gene, and the lowest M values correspond to the most stable expressed genes. The determination of the optimal number of reference genes for reliable normalization by geNorm relies on the calculation of the pairwise variation values (V), which measures pairwise variation (Vn/n + 1) between the sequentially ranked normalization factors NFn and NFn + 1, where *n* is the number of genes involved in the normalization factor. The recommended cut-off value^21^ below which there is no need for inclusion of another gene is 0.15.

The second tool used was NormFinder^51^ that requires the Cq values to be transformed to a linear scale expression quantity (without any negative values) by using the primer efficiency value **(E)**, where $\text{Linear quantity}\text{=}{\text{E}}^{-\text{Cq value }}.$ This software determines a stability value ranging from 0 to 1, where the lower values correspond to the best reference genes. Furthermore, this software provides additional information about the Intragroup and Intergroup variation. While intragroup variation describes how much the biological replicates in each life-cycle stage differ from each other, intergroup variation describes how much the averages of the replicates from a given life-cycle stage differ from the averages of other stages.

The third software used was RefFinder^52^ (http://blooge.cn/RefFinder/) that integrates four different software, namely BestKeeper and NormFinder, geNorm, and the Comparative delta-Ct method. RefFinder only requires inputting the Cq values from each sample and primers, and then it retrieves each software result and a comprehensive ranking gathering all results. RefFinder does not use the primer efficiency value in its data input; thus, each primer's corrected Cq values were submitted instead. The corrected Cq value is obtained with the following equation: Corrected Cq value = ${\text{Log}}_{2}{{\text{E}}}^{\text{Cq value}}$.

The ∆Ct method^96^ was used to determine if the reference genes most commonly used in the literature were differentially expressed among the six *S. mansoni* life-cycle stages, to calculate p14 differential expression in the pairing experiments, and to calculate EDD knockdown in the silencing experiments. For this, the geometric mean of the Cq values from the two best reference genes found in the present work (Smp_101310.1 and Smp_196510.1), measured across all samples, was used to normalize the expression of the genes of interest. Primers were retrieved from previous publications for Smp_316140 (p14)^97^ and Smp_165220 (EED)^28^.

### Statistical analyses

One-way ANOVA with Tukey correction for multiple comparisons was used to compare the differential expression across the *S. mansoni* stages of the eight reference genes frequently used in the literature. Unpaired Student t-test was used to compare the differential expression of Smp_316140 (p14) or of Smp_165220 (EED) across the pairing and silencing samples. GraphPad Prism software (version 8.0) was used to perform the analyses. p-value thresholds were *< 0.05, **< 0.01, ***< 0.001, and ****< 0.0001.

## Supplementary Information

Supplementary Information 1.

Supplementary Information 2.

Supplementary Information 3.
