# Supplementary material for: Assessment of reference genes at six different developmental stages of Schistosoma mansoni for quantitative RT-PCR
Source: Sci Rep. 2021 Aug 19;11:16816. doi: 10.1038/s41598-021-96055-7 (PMC8376997; doi:10.1038/s41598-021-96055-7)
Supplement: Supplementary file 1 — Supplementary Information 1. [file 41598_2021_96055_MOESM1_ESM.pdf]

Supplementary Figures for

Assessment of reference genes at six different developmental stages of *Schistosoma mansoni* for quantitative RT-PCR

Gilbert O. Silveira[1,2, #], Murilo S. Amaral[1, #], Helena S. Coelho[1], Lucas F. Maciel[1], Adriana S. A. Pereira[1,2], Giovanna G. O. Olberg[1], Patricia A. Miyasato[1], Eliana Nakano[1] & Sergio Verjovski-Almeida[1,2*]

[1] Laboratório de Parasitologia, Instituto Butantan, 05503-900, Sao Paulo, SP, Brazil.

[2] Departamento de Bioquímica, Instituto de Química, Universidade de São Paulo, 05508-900, Sao Paulo, SP, Brazil.

#Authors contributed equally

[*]Correspondence should be addressed to S.V.A. (email: verjo@iq.usp.br)

Supplementary Figures

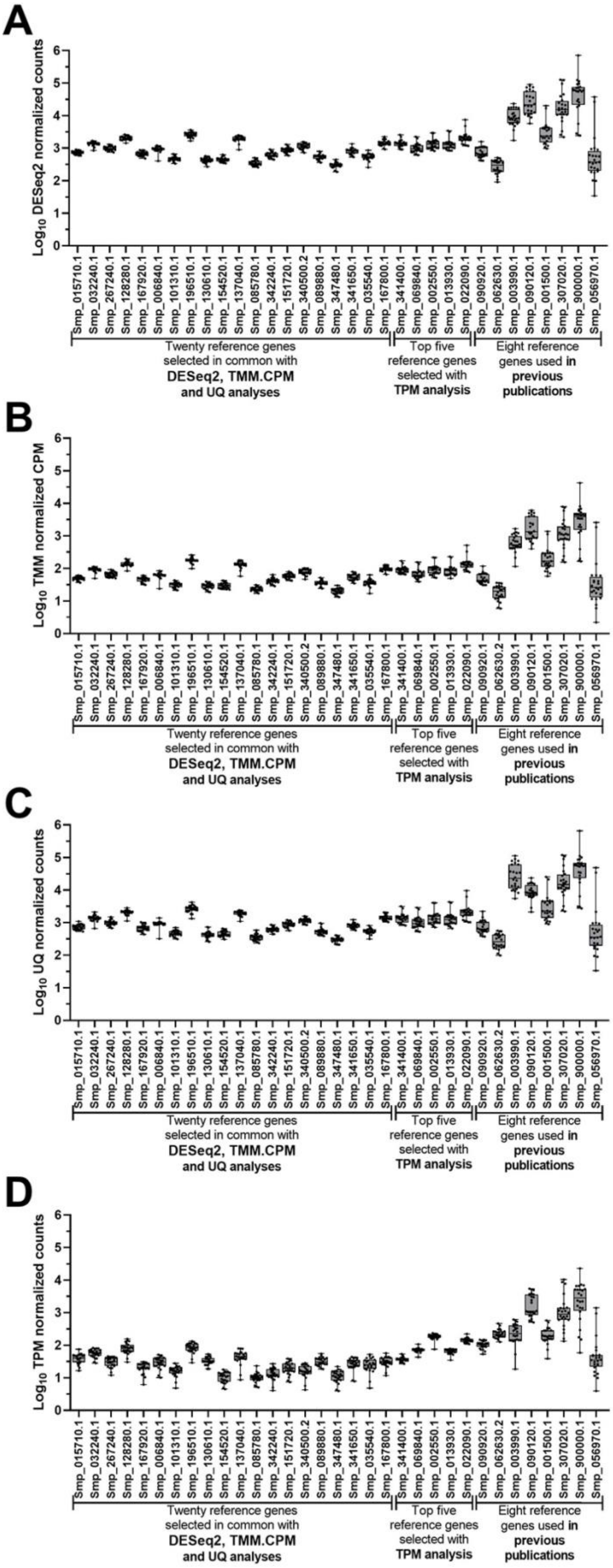
A
Log10 DESeq2 normalized counts
0
1
2
3
4
5
6
Smp_015710.1
Smp_032240.1
Smp_267240.1
Smp_128280.1
Smp_167920.1
Smp_006840.1
Smp_101310.1
Smp_196510.1
Smp_130610.1
Smp_154520.1
Smp_137040.1
Smp_085780.1
Smp_342240.1
Smp_151720.1
Smp_340500.2
Smp_089880.1
Smp_347480.1
Smp_341650.1
Smp_035540.1
Smp_167800.1
Smp_341400.1
Smp_069840.1
Smp_002550.1
Smp_013930.1
Smp_022090.1
Smp_090920.1
Smp_062630.1
Smp_003990.1
Smp_090120.1
Smp_001500.1
Smp_307020.1
Smp_900000.1
Smp_056970.1
Twenty reference genes selected in common with DESeq2, TMM.CPM and UQ analyses
Top five reference genes selected with TPM analysis
Eight reference genes used in previous publications
B
Log10 TMM normalized CPM
0
1
2
3
4
5
6
Smp_015710.1
Smp_032240.1
Smp_267240.1
Smp_128280.1
Smp_167920.1
Smp_006840.1
Smp_101310.1
Smp_196510.1
Smp_130610.1
Smp_154520.1
Smp_137040.1
Smp_085780.1
Smp_342240.1
Smp_151720.1
Smp_340500.2
Smp_089880.1
Smp_347480.1
Smp_341650.1
Smp_035540.1
Smp_167800.1
Smp_341400.1
Smp_069840.1
Smp_002550.1
Smp_013930.1
Smp_022090.1
Smp_090920.1
Smp_062630.2
Smp_003990.1
Smp_090120.1
Smp_001500.1
Smp_307020.1
Smp_900000.1
Smp_056970.1
Twenty reference genes selected in common with DESeq2, TMM.CPM and UQ analyses
Top five reference genes selected with TPM analysis
Eight reference genes used in previous publications
C
Log10 UQ normalized counts
0
1
2
3
4
5
6
Smp_015710.1
Smp_032240.1
Smp_267240.1
Smp_128280.1
Smp_167920.1
Smp_006840.1
Smp_101310.1
Smp_196510.1
Smp_130610.1
Smp_154520.1
Smp_137040.1
Smp_085780.1
Smp_342240.1
Smp_151720.1
Smp_340500.2
Smp_089880.1
Smp_347480.1
Smp_341650.1
Smp_035540.1
Smp_167800.1
Smp_341400.1
Smp_069840.1
Smp_002550.1
Smp_013930.1
Smp_022090.1
Smp_090920.1
Smp_062630.2
Smp_003990.1
Smp_090120.1
Smp_001500.1
Smp_307020.1
Smp_900000.1
Smp_056970.1
Twenty reference genes selected in common with DESeq2, TMM.CPM and UQ analyses
Top five reference genes selected with TPM analysis
Eight reference genes used in previous publications
D
Log10 TPM normalized counts
0
1
2
3
4
5
6
Smp_015710.1
Smp_032240.1
Smp_267240.1
Smp_128280.1
Smp_167920.1
Smp_006840.1
Smp_101310.1
Smp_196510.1
Smp_130610.1
Smp_154520.1
Smp_137040.1
Smp_085780.1
Smp_342240.1
Smp_151720.1
Smp_340500.2
Smp_089880.1
Smp_347480.1
Smp_341650.1
Smp_035540.1
Smp_167800.1
Smp_341400.1
Smp_069840.1
Smp_002550.1
Smp_013930.1
Smp_022090.1
Smp_090920.1
Smp_062630.2
Smp_003990.1
Smp_090120.1
Smp_001500.1
Smp_307020.1
Smp_900000.1
Smp_056970.1
Twenty reference genes selected in common with DESeq2, TMM.CPM and UQ analyses
Top five reference genes selected with TPM analysis
Eight reference genes used in previous publications

Figure S1. Expression patterns of candidate reference genes at different *Schistosoma mansoni* life-cycle stages. Twenty-five candidate reference genes that have the lowest coefficients of variation of their expression values across 24 RNA-Seq libraries were selected in this work for evaluation of their stability by RT-qPCR assays using six different *S. mansoni* life-cycle stages (see Methods). The libraries used in the analysis are described in Supplementary Table S1. Eight candidate reference genes commonly used in previous publications were also selected. Log_{10} transformed expression profiles from all selected reference genes are represented by their minimum to maximum values for each of the four RNA-Seq normalization methods tested: **(A)** DESeq2; **(B)** TMM.CPM; **(C)** UQ; and **(D)** TPM. The horizontal line represents the median, and the boxes and whiskers represent the inter-quantile and min to max ranges, respectively.

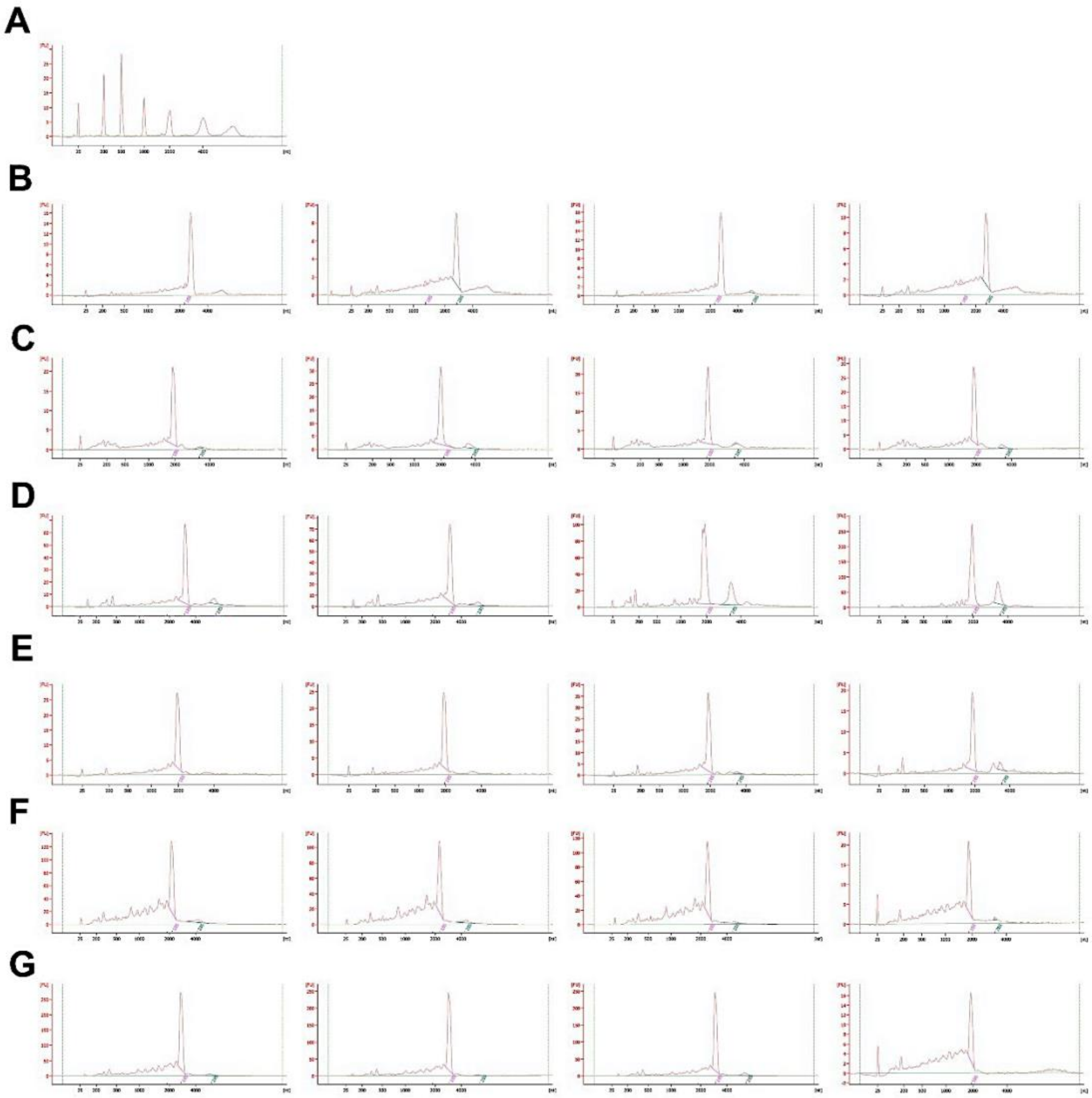

Figure S2. RNA integrity measurement with the Agilent RNA 6000 Pico Kit in a 2100 Bioanalyzer Instrument for all the life cycle stage samples used for RT-qPCR assays. **(A)** Ladder run, **(B)** Eggs samples, **(C)** Miracidia samples, **(D)** Cercariae samples, **(E)** 48-h-schistosomula samples, **(F)** Adult males samples and **(G)** Adult females samples. The y-axis represents the fluorescence intensity for each of the graphs, while the x-axis represents the run time. All four biological replicates of each life cycle stage sample are represented side by side. A typical single peak (denoting 18S ribosomal subunit RNA) is expected.

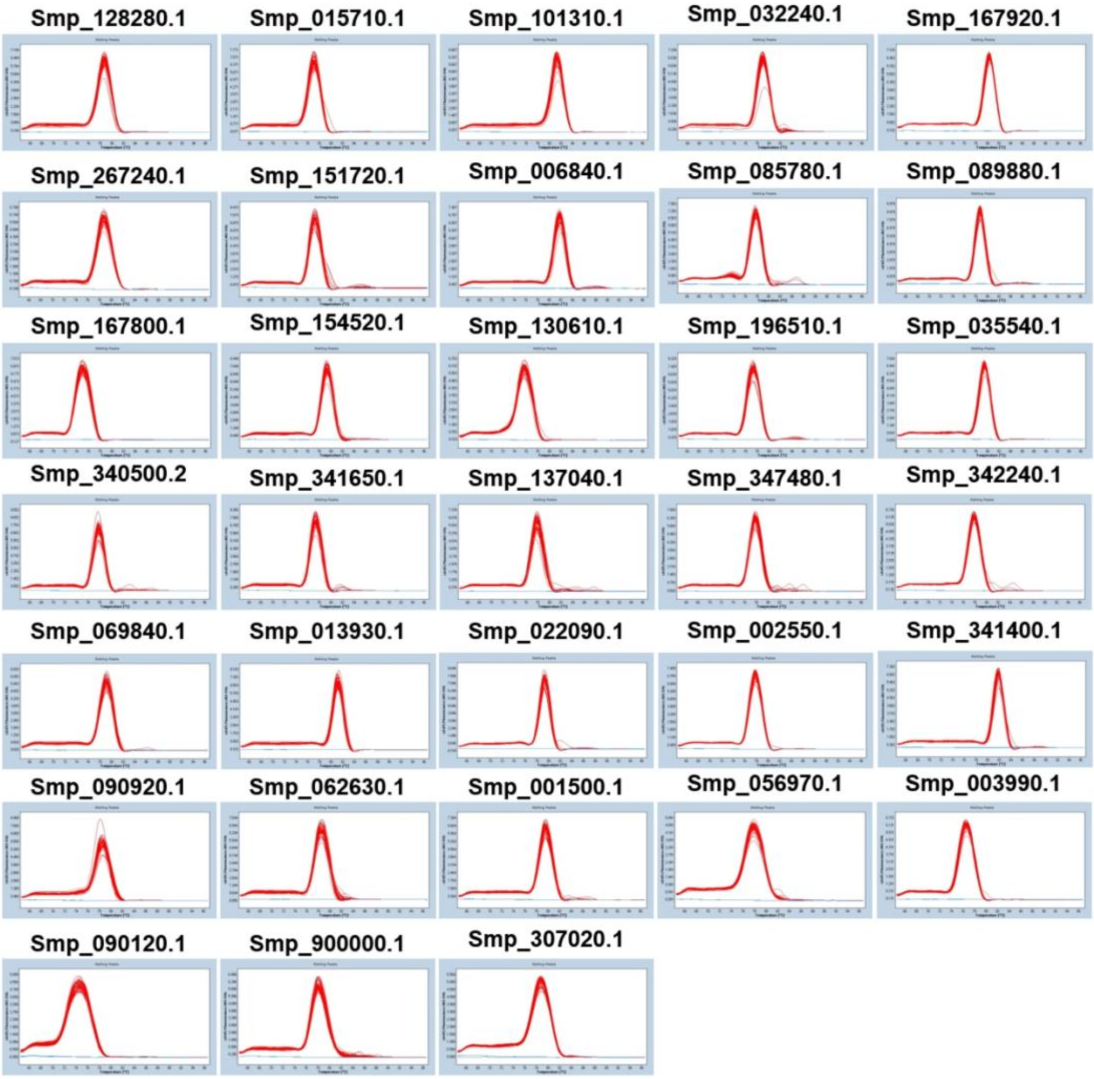

Figure S3. Melting curves for all amplicons obtained in the RT-qPCR assays. For each of the graphs, the y-axis represents the fluorescence intensity while the x-axis represents temperature. Smp_nnnnnn is the code for each of the genes selected for RT-qPCR normalization in different life-cycle stages of *S. mansoni*. The sequences of the primer pairs used for each gene are shown in Supplementary Table S7.

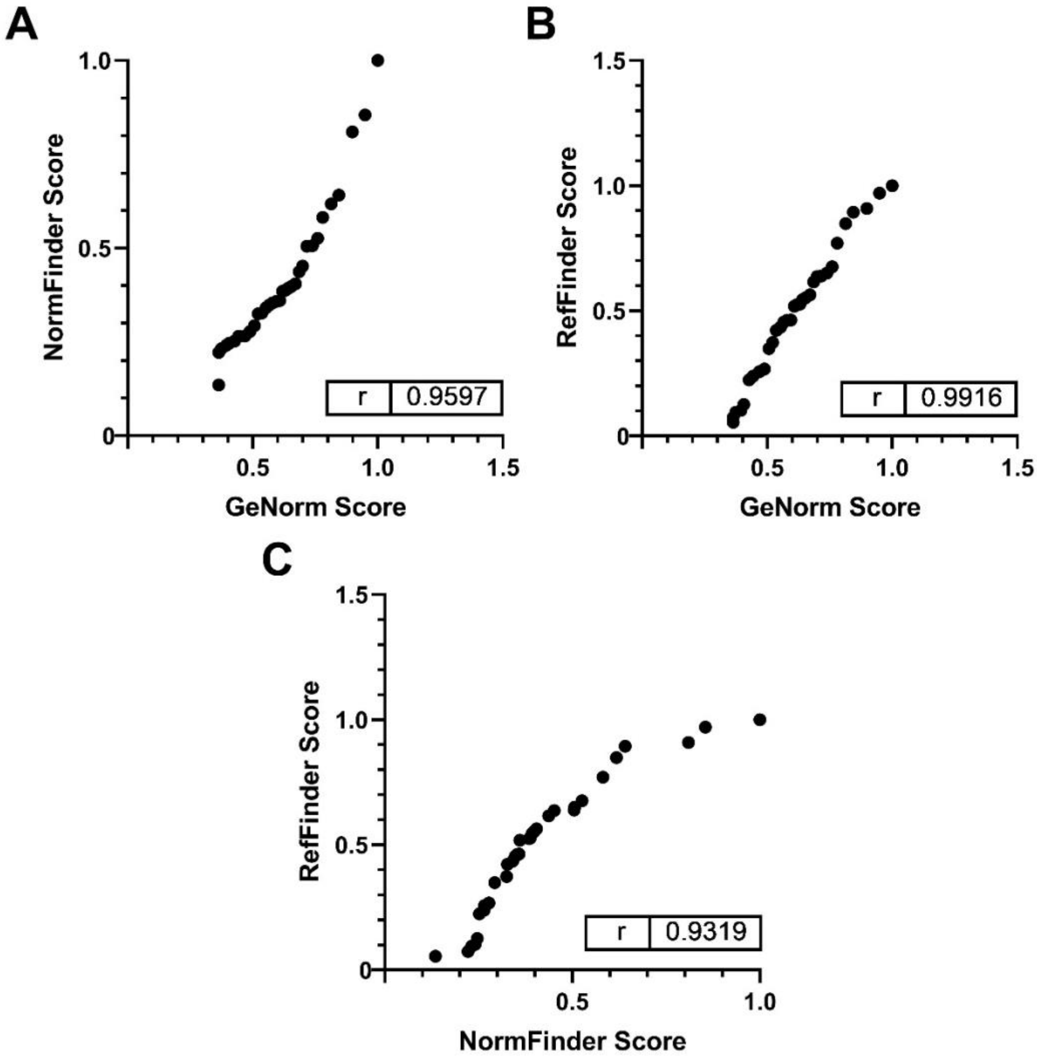

Figure S4. Pearson correlation calculated between the results of GeNorm, NormFinder, and RefFinder. The stability scores for each of the candidate reference genes obtained from the three different software (GeNorm, NormFinder, and RefFinder) were used to calculate the Pearson correlation coefficient r. In **(A)** correlation between the results from GeNorm and NormFinder, in **(B)** correlation between GeNorm and RefFinder, and in **(C)** correlation between RefFinder and NormFinder results. Each dot represents one of the 33 candidate reference genes tested. For **(A)**, **(B)** and **(C),** p-value <0.001.

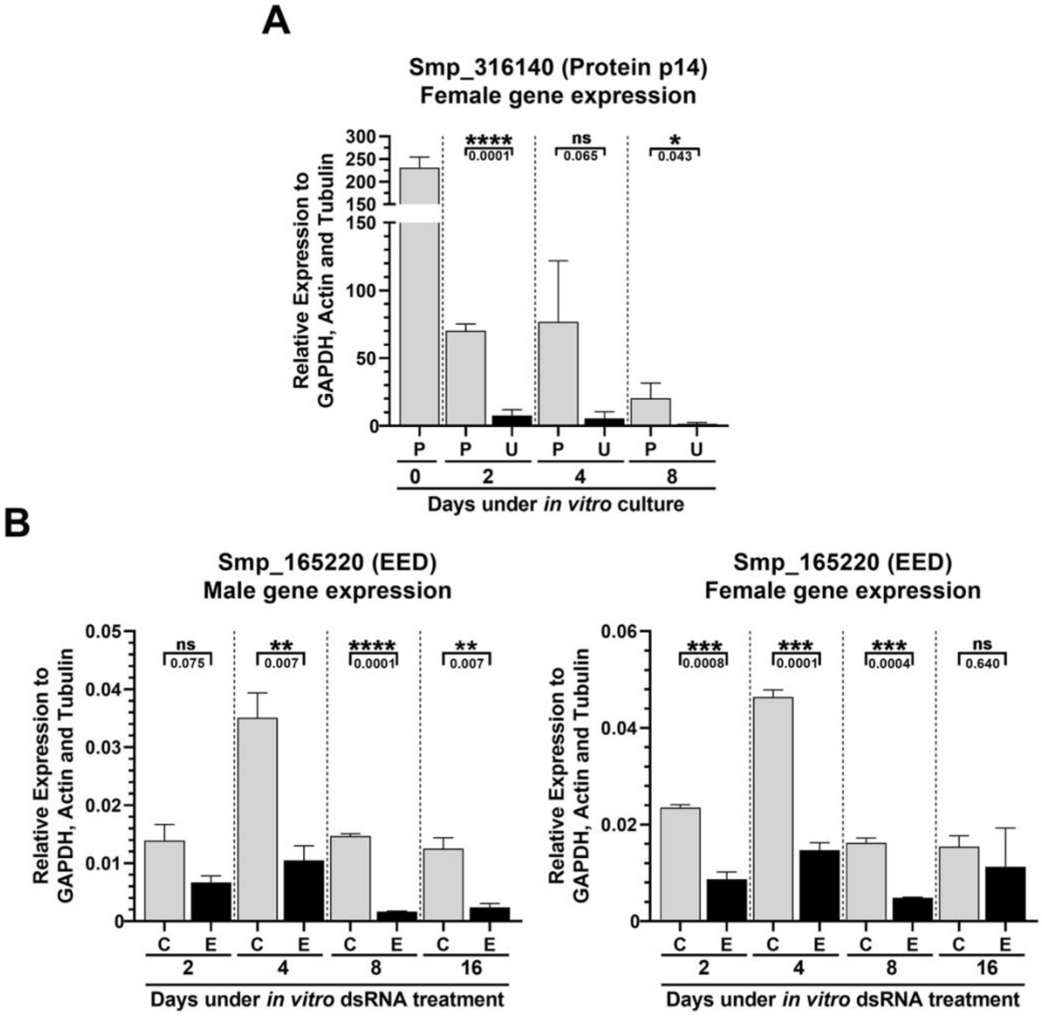

Figure S5. Relative expression of two different protein coding genes in *S. mansoni* under several culturing conditions, normalized by three reference genes found in the literature. **(A)** Female adult worm gene expression pattern of Smp_316140 gene (Protein p14) across different *in vitro* culturing conditions. Quantitative RT-qPCR was performed with RNA samples from females that were paired (P) or unpaired (U) to males and cultivated *in vitro* for 2, 4 or 8 days. Day 0 stands for paired females retrieved right after perfusion. **(B)** Male and female adult worm Smp_165220 (*EED*) gene expression in samples obtained from couples treated with dsRNA targeting either the control unrelated dsmCherry gene (C) or the Smp_165220 (*EED*) gene (E) *in vitro* for 2, 4, 8 or 16 days. The expression values are represented as the relative expression using as normalizer the geometric mean of three commonly used reference genes, Smp_056970 (*GAPDH*), Smp_307020 (*Actin*) and Smp_090120 (*α-tubulin*). Bars represent the standard deviation of the mean from three biological replicates for each experiment. Three technical replicates were assayed for each of the three biological replicates. Unpaired student t-test was used to calculate the statistical significance of the expression differences in the comparisons (* p-value ≤ 0.05; ** p-value ≤ 0.01; *** p-value ≤ 0.001; **** p-value ≤ 0.0001; ns = p-value > 0.05). The p-value obtained from the Student t-test is represented under the brackets.
